# Pre- and postsynaptic nanostructures increase in size and complexity after induction of long-term potentiation

**DOI:** 10.1016/j.isci.2023.108679

**Published:** 2023-12-07

**Authors:** Valérie Clavet-Fournier, ChungKu Lee, Waja Wegner, Nils Brose, JeongSeop Rhee, Katrin I. Willig

**Affiliations:** 1 Group of Optical Nanoscopy in Neuroscience, Max Planck Institute for Multidisciplinary Sciences, Göttingen, Germany; 2 Göttingen Graduate Center for Neurosciences, Biophysics, und Molecular Biosciences (GGNB), Göttingen, Germany; 3 Department of Molecular Neurobiology, Synaptic Physiology Group, Max Planck Institute for Multidisciplinary Sciences, Göttingen, Germany; 4 Center for Nanoscale Microscopy and Molecular Physiology of the Brain, University Medical Center Göttingen, Göttingen, Germany; 5 Department of Molecular Neurobiology, Max Planck Institute for Multidisciplinary Sciences, Göttingen, Germany

**Keywords:** Neuroscience, Sensory neuroscience

## Abstract

Synapses, specialized contact sites between neurons, are the fundamental elements of neuronal information transfer. Synaptic plasticity involves changes in synaptic morphology and the number of neurotransmitter receptors, and is thought to underlie learning and memory. However, it is not clear how these structural and functional changes are connected. We utilized time-lapse super-resolution STED microscopy of organotypic hippocampal brain slices and cultured neurons to visualize structural changes of the synaptic nano-organization of the postsynaptic scaffolding protein PSD95, the presynaptic scaffolding protein Bassoon, and the GluA2 subunit of AMPA receptors by chemically induced long-term potentiation (cLTP) at the level of single synapses. We found that the nano-organization of all three proteins increased in complexity and size after cLTP induction. The increase was largely synchronous, peaking at ∼60 min after stimulation. Therefore, both the size and complexity of individual pre- and post-synaptic nanostructures serve as substrates for tuning and determining synaptic strength.

## Highlights

• Time-lapse STED microscopy of structural synaptic changes after cLTP induction
• PSD95 nano-organizations increase slower than spine heads after cLTP induction
• Synchronous growth of presynaptic Bassoon, and postsynaptic PSD95 and AMPAr
• PSD95 and Bassoon nanostructures increase in complexity after stimulation

## Graphical abstract

**Figure:**
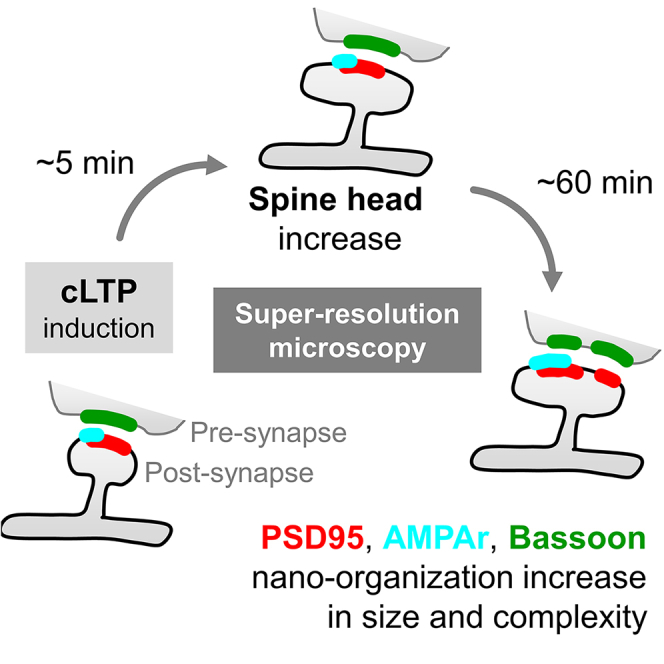

## Introduction

Chemical synapses, which consist of presynaptic and postsynaptic compartments as well as a synaptic cleft, employ a multitude of exquisitely arranged and coordinated proteins to convert electrical into chemical signals, and vice versa. The rate and efficacy of synaptic transmission varies among synapses, and is thus unique to each synapse. This diversity is in large part due to differences in release + probability of synaptic vesicles at the presynapse and the heterogeneous distribution of neurotransmitter receptors at the postsynapse. However, whether and how synaptic strength correlates with changes in the structural organization of synapses and their protein components remains controversial. Decades ago it was shown by electron microscopy that the postsynaptic density (PSD), a proteinaceous specialization of excitatory postsynapses is not always continuous but often disrupted or perforated. Such perforations occur mainly on larger postsynaptic spines, specialized dendritic protrusions of excitatory glutamatergic postsynapses, and can be of various, complex shape.^1^ The proportion of perforated synapses and their size were shown to increase with development or the induction of long-term potentiation (LTP).^2^^,^^3^ Recently, such rapid structural changes of the PSD and non-synaptic compartments were quantified in detail after the induction of LTP in single spines.^2^ While synaptic perforations are too small to be visible by conventional light microscopy, the application of super-resolution techniques enables their visualization in living cells, even in the intact brain of a living mouse. We and others showed that the postsynaptic density protein PSD95, a scaffolding protein highly enriched in excitatory postsynapses, is also not always assembled in a continuous structure but in nanoclusters or more complex shapes^3^^,^^4^^,^^5^; these assemblies change their morphology at baseline *in vivo* on the timescale of minutes to hours^5^ and increase in size upon enhanced activity in an enriched environment.^6^ While spines increase rapidly in volume after LTP induction, PSD95 assemblies increase in size much more slowly, over a few hours.^7^^,^^8^ The recent discovery that components of the presynaptic active zone align with proteins of the postsynaptic density and glutamate receptors in so-called nanocolumns^9^^,^^10^ intensified the investigation of the structural nano-organization of the synapse and its functional significance.

However, the corresponding studies have several caveats. First, studies using conventional two-photon microscopy are limited in resolution and cannot resolve the PSD95 nanostructure. Second, most studies using super-resolution microscopy to address the synaptic nano-organization of PSD95 assessed only the size and/or number of clusters, so called nano-clusters, nano-domains, or nano-modules.^3^^,^^4^^,^^9^^,^^10^ However, with *in vivo* STED microscopy of an adult PSD95-eGFP knock-in mouse we found that endogenous PSD95 is nano-organized in various shapes, such as perforated, ring-like, or more complex structures.^5^ This is consistent with results from electron microscopy.^2^ Thus, a simple cluster analysis alone misses key features required for our understanding of the functional role of the complex synaptic nanostructure. Third, studies of activity-dependent changes in the synaptic nano-organization usually compare different datasets collected at different time points after stimulation and are therefore indirect measurements; this holds true for super-resolution imaging^9^^,^^11^ and electron microscopy.^2^

Here, we overcome these limitations by employing time-lapse super-resolution STED microscopy of endogenous PSD95 in living organotypic hippocampal slices. We investigated the plasticity of spines and endogenous PSD95 nano-organization upon NMDA dependent chemical LTP (cLTP) and assessed changes in size and nanostructure similar to a recent electron microscopic study.^2^ PSD95 nano-organizations increased in complexity and size after cLTP induction, with the restructuring of PSD95 assemblies occurring more slowly than the rapid growth of the spine head. Furthermore, we tested whether arrangements of presynaptic scaffolds and glutamate receptors are equally complex in nanostructure and dynamics. We found a strong correlation between the nano-organization of presynaptic Bassoon and postsynaptic PSD95; Bassoon clusters are similarly complex in structure and increase in size by a similar factor upon cLTP induction. In contrast, AMPA receptor (AMPAr) nanoclusters containing the GluA2 subunit do not show a complex structure. cLTP induction resulted in an increase of synaptic AMPAr nanocluster size and number, contradicting a purely modular composition previously suggested.^11^ That is, the change in the AMPAr-induced excitatory postsynaptic current (EPSC) size was more sensitive and rapid than the dynamics of AMPAr nanoclusters, which was similar to the slow change in PSD95 assemblies.

## Results

### Time-lapse stimulated emission depletion imaging of endogenous PSD95 in organotypic hippocampal slices

**Figure 1:**
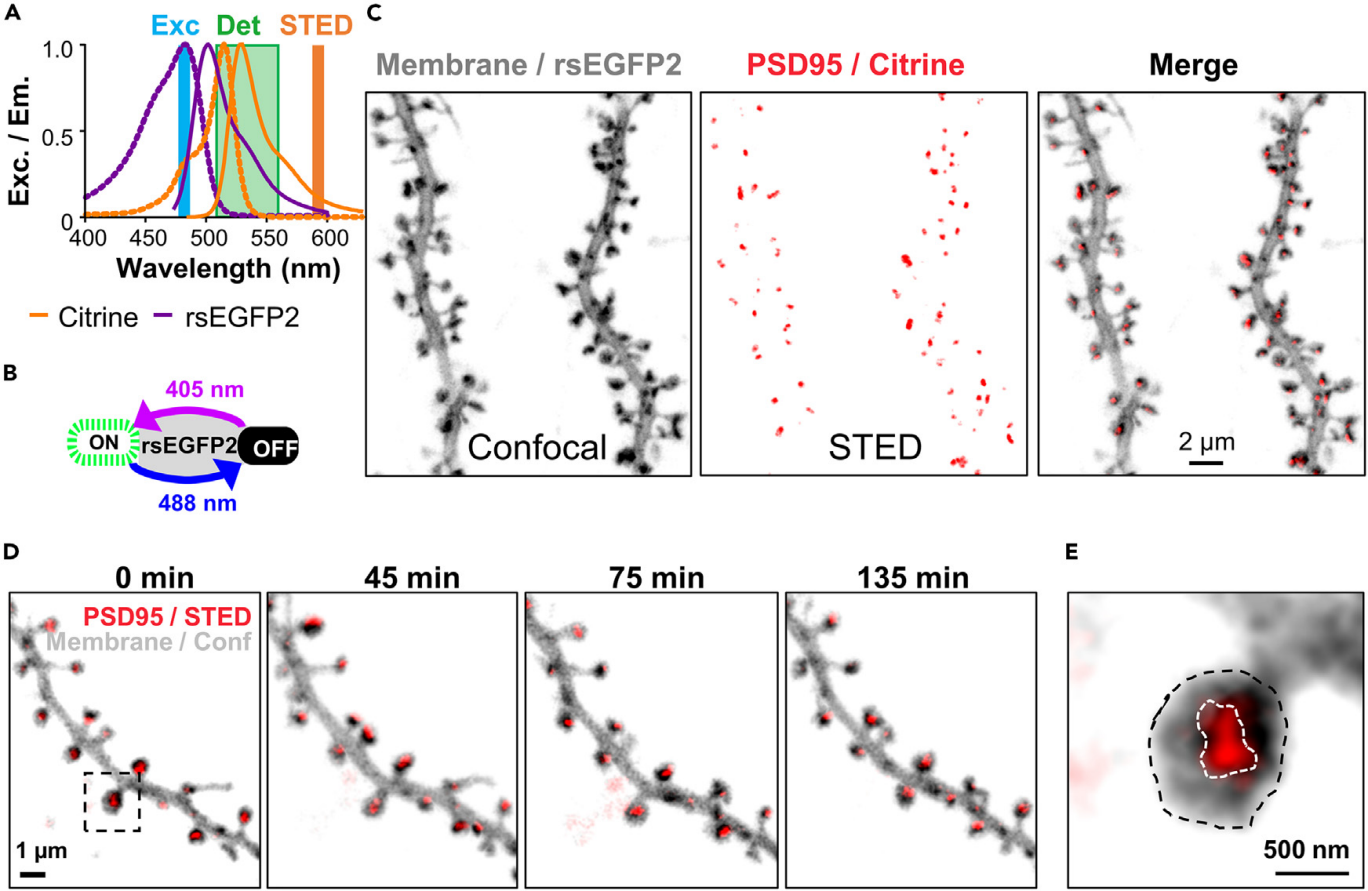
Super-resolution microscopy of endogenous PSD95 in hippocampal organotypic slices (A and B) Dual-label schema by sequential readout: The FPs Citrine and rsEGFP2 are excited with blue light (Exc) and detected between 510 and 560 nm (Det); stimulated emission depletion (STED) is performed on demand at 595 nm (A). The reversibly switchable FP rsEGFP2 is switched to the on state with UV light at 405 nm and to the off state with blue light at 488 nm. (C) Hippocampal neurons express a myristoylation tag (myr) and a dendrite targeting sequence (LDLR) fused to rsEGFP2 to label the dendritic membrane and an antibody-like tag (PSD95.FingR) fused to Citrine to label endogenous PSD95. Super-resolution STED microscopy of PSD95 (red) and confocal imaging of the neuronal membrane (gray). (D) Time-lapse imaging of PSD95 and spine morphology over >2 h. (E) Magnification of boxed area in (D); encircling of the PSD95 assembly and spine head for size analysis. Images are smoothed and maximum intensity projection (C, D).

To assess the morphology and nano-plasticity of PSD95, we used a home-built combined STED and confocal microscope^12^^,^^13^ to image live organotypic hippocampal slices. Endogenous PSD95 was labeled with a transcriptionally regulated recombinant antibody-like protein termed FingR,^6^^,^^14^ fused to the fluorescent protein Citrine. Additionally, we expressed a myristoylation (myr) motif to tag the neuronal membrane with the reversibly switchable fluorescent protein rsEGFP2.^13^ Both constructs were expressed via the transduction of recombinant adeno-associated viral particles (AAV) into the CA1 region of the hippocampus. rsEGFP2 emits fluorescence in a similar spectral range as EGFP and thus very close to Citrine. Separation between Citrine and rsEGFP2 fluorescence was achieved in time by switching rsEGFP2 on and off; both fluorescent proteins (FP) were excited with blue light and detected in the same broad spectral window (Figure 1A).^15^ rsEGFP2 was recorded with additional 405 nm light that switched rsEGFP2 to the one state while recording the image (Figure 1B). Recording without 405 nm light switched the rsEGFP2 to the off state and thus the pure PSD95 image was recorded (Figure S1). In this manner we imaged the PSD95 and the dendritic membrane in sequential images directly one after the other. The image of the membrane also included the PSD95 signal as Citrine is not switchable; this was negligible due to the lower signal of PSD95 compared to that of the membrane. The STED beam was switched on only in the Citrine image to record the nano-organization in super-resolution; spine morphology was recorded without the STED beam in confocal mode, as this was sufficient to determine the size of the spine heads and to reduced the overall photo bleaching (Figure 1C). Recording images at different time points revealed the structural changes of the PSD95 nano-organization (Figure 1D). Sizes were analyzed by encircling the spine head and PSD95 assemblies (Figure 1E).

### PSD95 assembly size, spine heads and synaptic strength increase upon chemically induced long-term potentiation

**Figure 2:**
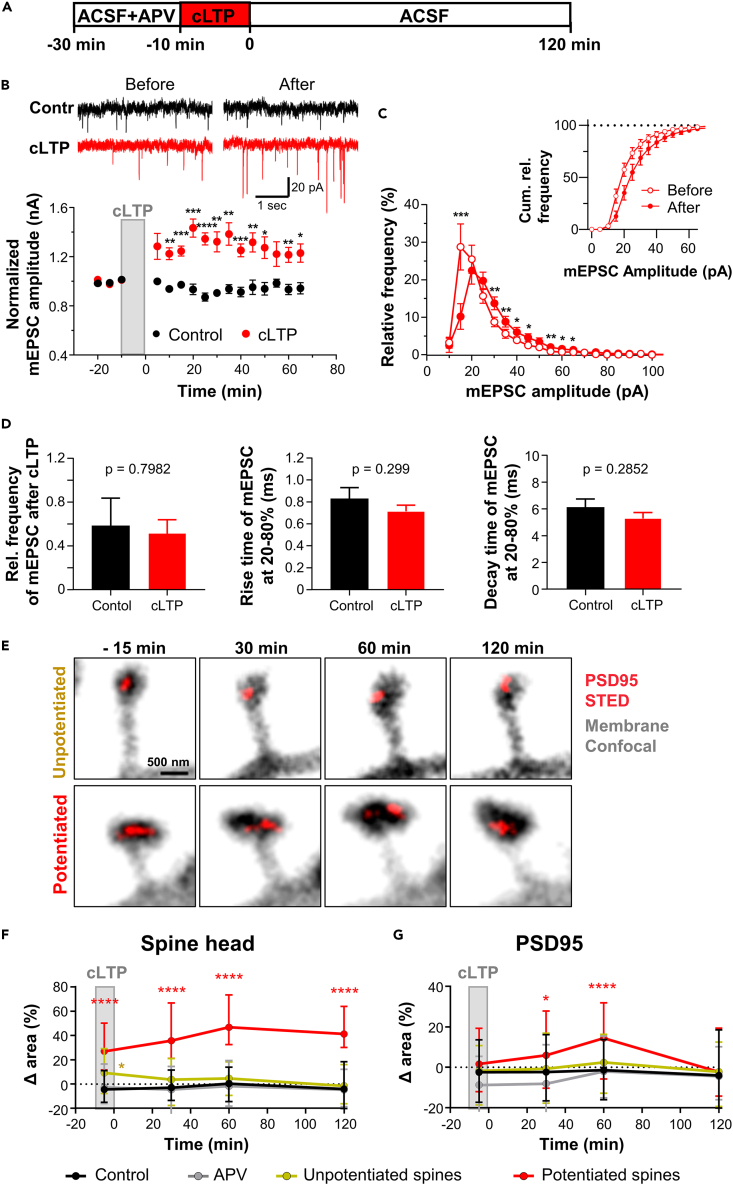
Increase in synaptic strength, spine head and PSD95 assembly size after cLTP induction (A) Time-line of the experiment. Chemical LTP (cLTP) is induced by ACSF containing zero Mg^2+^, 200 μM glycine, 20 μM bicuculline. (B–D) Whole cell voltage-clamp recording of mEPSC in organotypic hippocampal slices. (B) Representative mEPSC traces (top) of CA1 pyramidal neurons before (−15 min) and after (+65 min) cLTP induction and averaged normalized current ±SEM (bottom); control without cLTP (black) and with cLTP induction (red) (unpaired t-test; ∗p < 0.05; ∗∗p < 0.01; ∗∗∗p < 0.001, ∗∗∗∗p < 0.0001). (C) Frequency distribution of mEPSC amplitude and cumulative frequency before (open circle, −15 min) and after cLTP induction (closed circle, 65 min) ±SEM (paired t-test, before: after). (D) Normalized frequencies of mEPSC after 65 min compared to before cLTP induction (left), rise time (middle) and decay times (right) for 20–80% of mEPSC. Bars represent average ±SEM with (red) and without (black) induction of cLTP; no significant difference between cLTP and control (unpaired t-test) (E) Representative images of potentiated and unpotentiated spines before and after cLTP induction. (F) Median and interquartile range (IQR; 25% and 75% percentile) of changes in spine head area of potentiated and unpotentiated spines after cLTP relative to control. Control conditions were continuously kept in ACSF (control) or spines at blocked activity measured in ACSF containing APV (APV). Changes were compared to control for each time-point (Data did not pass D'Agostino-Pearson normality test; Kruskal-Wallis with Dunn’s multiple comparisons test; −5 min: Pot vs. Ctr, p < 0.0001; Unpot vs. Ctr, p = 0.02; APV vs. Ctr, p > 0.99; 30 min: Pot vs. Ctr, p < 0.0001; Unpot vs. Ctr, p = 0.27; APV vs. Ctr, p = 0.84; 60 min: Pot vs. Ctr, p < 0.0001; Unpot vs. Ctr, p = 0.13; APV vs. Ctr, p > 0.99; 120 min: Pot vs. Ctr, p < 0.0001; Unpot vs. Ctr, p > 0.99; APV vs. Ctr, p > 0.99). (G) Median and IQR for PSD95 area of the spines analyzed in (F) (Data did not pass D'Agostino-Pearson normality test; Kruskal-Wallis with Dunn’s multiple comparisons test; −5 min: Pot vs. Ctr, p = 0.32; Unpot vs. Ctr, p > 0.99; APV vs. Ctr, p = 0.18; 30 min: Pot vs. Ctr, p = 0.02; Unpot vs. Ctr, p > 0.99; APV vs. Ctr, p = 0.17; 60 min: Pot vs. Ctr, p < 0.0001; Unpot vs. Ctr, p = 0.14; APV vs. Ctr, p > 0.99; 120 min: Pot vs. Ctr, p = 0.74; Unpot vs. Ctr, p > 0.99; APV vs. Ctr, p > 0.99). (B–D) Number of recorded cells: cLTP: 9; control: 5. (F, G) Number of analyzed spine changes at −5, 30, 60, 120 min: Control: 121, 221, 220, 100; APV:, 153, 227, 198, 82; unpotentiated: 66, 202, 196, 130; potentiated: 46, 106, 110, 63. Number of analyzed PSD95 assembly changes at the same time points: Control: 119, 220, 212, 92; APV: 153, 222, 167, 70; unpotentiated: 68, 201, 195, 123; potentiated: 45, 101, 108, 59. Number of hippocampal slices, one dendrite/neuron per slice: Control: 10; APV: 13; unpotentiated: 19; potentiated: 19. Source data: Table S1.

To further explore structural and functional synaptic plasticity, we induced cLTP by adding 200 μM glycine and 20 μM bicuculline to ACSF without Mg^2+^ ions (Figure 2A). Before cLTP induction, the organotypic hippocampal slices were kept in ACSF solution containing APV to block basal neuronal activity induced by NMDA receptors for 20 min. To assess the functional changes of individual synapses induced by cLTP we recorded spontaneous excitatory events by whole cell voltage-clamp recordings in pyramidal neurons of CA1,^16^^,^^17^^,^^18^ with the rationale that the amplitude and kinetics of corresponding miniature excitatory postsynaptic currents (mEPSC) reflect, in part, functional and structural changes occurring at synapses. mEPSCs were measured for up to 1 h after cLTP induction, while all solutions contained TTX to avoid effects caused by propagating action potentials. mEPSC amplitudes increased immediately after cLTP induction by a factor of 1.28 ± 0.05 (mean ± SEM) and enhanced currents were maintained for at least 60 min; without stimulation mEPSC amplitudes did not increase (0.93 ± 0.03; control) (Figure 2B).

To determine whether cLTP is a phenomenon that affects only a limited number or specific synapses, we further analyzed the frequency distribution of mEPSC amplitudes. After cLTP, the distribution of mEPSC amplitudes shifted to the right and widened slightly (Figure 2C). A plot of the normalized frequencies reveals a very similar shape of mEPSC amplitudes before and after cLTP induction (Figure S2) which indicates that the vast majority of synapses increase in strength by a similar factor. In contrast, a mere broadening of the mEPSC amplitude distribution, or in the extreme case, a splitting into two peaks would indicate the emergence of new active synapses or an increase in synaptic strength solely in a subset of synapses.

The relative changes in mEPSC frequency showed no difference between the control (0.52 ± 0.13) and cLTP-induced groups (0.58 ± 0.26) (Figure 2D, left). This indicates that the increase in mEPSC amplitude is not due to an increase in synaptic vesicle release machinery, but rather to changes in the number of receptors at the post-synapse. To assess the kinetics of the mEPSC, we analyzed the 20–80% rise time and the decay time of mEPSC events before and after cLTP induction. The rise time was not significantly different between cLTP (0.83 ± 0.1 ms) and control (0.71 ± 0.05 ms) groups (Figure 2D, middle). Likewise, no difference in the decay time was found between cLTP (5.3 ± 0.4 ms) and control groups (6.2 ± 0.6 ms) (Figure 2D, right).

To investigate structural synaptic plasticity after cLTP induction we performed time-lapse STED and confocal imaging as described above. After the acquisition of a baseline image, cLTP was induced for 10 min before the perfusion was switched to standard ACSF. Image stacks of the PSD95 labeling and spine membrane were acquired ∼5 min before cLTP (t = −15 min), at 5 min during cLTP induction (t = −5 min) and 30, 60, and 120 min after cLTP induction. We imaged each field of view a maximum of four times to reduce photo bleaching. Therefore, we randomly imaged in two groups either before cLTP and at −5, 30 and 60 min or before cLTP and at 30, 60 and 120 min after cLTP induction. Spines that were within the z stack at all time points of the measurement series were analyzed for spine head and PSD95 assembly size. Spines that underwent a persistent enlargement of ≥15% at 60 min and 120 min after cLTP induction were considered potentiated spines. All other spine heads, i.e., with <15% enlargement, were regarded unpotentiated. Figure 2E shows a representative example of a potentiated and unpotentiated spine. On average, ∼40% of the spine heads showed a persistent enlargement of at least 15% at 60 min and 120 min following cLTP induction (111 of 279 spines in 19 cells); spines undergoing an enlargement (potentiated spines) were smaller than those not undergoing an enlargement (unpotentiated spines) (Figure S3A). Dendrites with less than 20% of enlarged spines were discarded. Plotting the changes in spine head size at the different time points shows a significant increase in median in potentiated spines already during the cLTP induction (Figure 2F; single spine traces Figure S3B) by 27 (interquartile range (IQR), 12–50) % compared to controls, as shown previously.^7^^,^^8^ This enlargement increased even further up to 47 (IQR 33–73) % after 60 min and 41 (IQR 30–64) % after 120 min. Unpotentiated spine heads, in turn, showed only a small but significant median increase of 9 (IQR -8 – +29) % during cLTP induction compared to control, which was not sustained for the rest of the time course. All changes were assessed in relation to changes of a baseline control condition, which was measured in ACSF without stimulation. Blocking of NMDA receptors with APV during the whole-time course prevented changes in spine head size (Figure 2F).

The size of PSD95 assemblies on potentiated spines was larger than the controls 30 and 60 min after cLTP induction (Figures 2G and S3B). However, with a median of 6 (IQR -10 – +28) % at 30 min and 14 (IQR -6 – +32) % at 60 min, this increase was less than the increase in spine head size. Such a delayed increase in PSD95 cluster size vs. spine head size had also been shown in a previous study.^7^

A plot of changes in the size of the spine head or PSD95 assemblies at 60 min after cLTP induction versus their original size (Figures S4A and S4B) shows that small spines or PSD95 assemblies tend to increase, while large spines or PSD95 assemblies decrease in the control groups (control, APV, unpotentiated). This is a very common statistical effect termed regression to the mean; it is crucial to keep a population stable at a constant mean and variance. The increase in size after potentiation is reflected in the higher regression line for potentiated spines compared to unpotentiated spines (Figure S4A, red). Furthermore, the slope changes from negative for unpotentiated to positive for potentiated spines, suggesting that the increase in size is multiplicative; it depends on the original size of the spine. For PSD95 assembly sizes, however, we observed only a slightly higher regression line for potentiated spines (Figure S4B).

### Restructuring of the PSD95 nano-pattern after induction of chemically induced long-term potentiation

**Figure 3:**
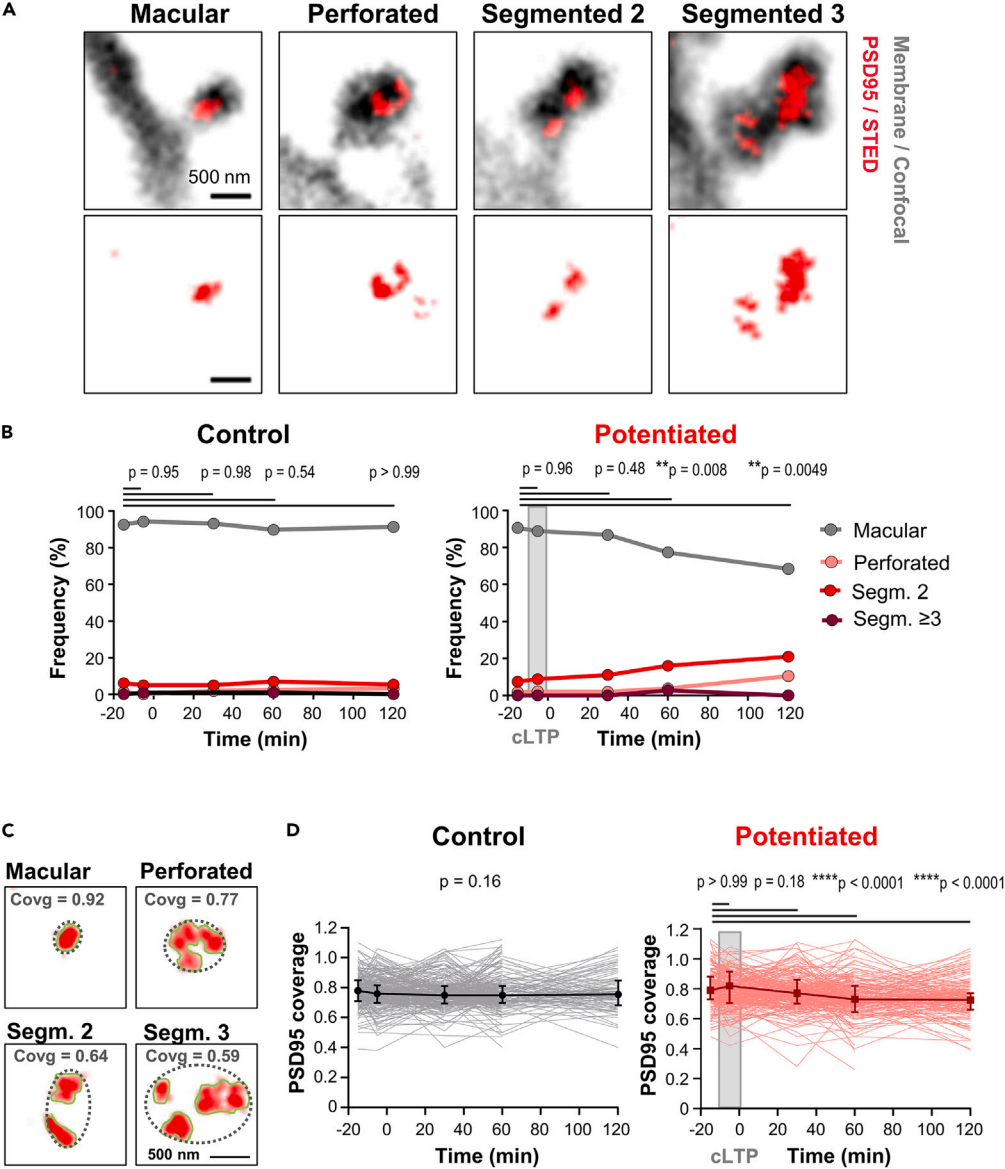
Reorganization of the PSD95 nano-pattern after cLTP induction (A) STED images of PSD95 (red) and confocal images of the spine membrane (gray) depicting representative examples of macular or perforated PSD95 assemblies and such consisting of 2 or 3 segments. (B) Percentage of macular, perforated, segmented 2, and segmented ≥3 PSD95 assemblies per spine for up to 120 min after cLTP induction (right) or control without stimulation (left) (Mixed-effects analysis with Dunett’s multiple comparisons test). Source data: Table S2. (C) The PSD95 coverage (covg) ratio was calculated by dividing the area covered with PSD95 (red) by the greatest extent of the synapse (dashed black ellipse). (D) PSD95 coverage ratio for control and cLTP potentiated spines over a 120 min time course; single spine traces in light colors overlaid with median (error bars represent IQR; mixed-effects analysis with Dunett’s multiple comparisons test; overall p value (control) and p value after multiple comparisons test (potentiated)). Source data: Table S3. (B, D) Number of analyzed PSD95 assemblies at −15, −5, 30, 60, 120 min: control: 227, 120, 221, 213, 92; potentiated: 109, 45, 102, 109, 60.

In large spine heads, PSD95 is often organized into very complex structures that include perforations, U-shapes, or more complex shapes; it appears in single or in multiple patches (Figure 3A). We have observed a similar PSD95 nano-pattern in a knock-in reporter mouse line^19^ and also *in vivo* with the same FingR.PSD95 construct that we used in the present study.^6^ Similar shapes were also observed with electron microscopy of post-synaptic densities.^20^ We categorized the PSD95 nanoarchitecture as follows:^20^ (1) Simple PSD95 assemblies without a perforation were assigned a macular shape; (2) assemblies with a hole, U-shape or more complex shape were assigned a perforated shape; (3) two separated PSD95 spots were assigned segmented 2; (4) three or more separated PSD95 spots were classified as segmented ≥3. Segmented spots were not further classified by their shape. We performed this classification for spines before, during and after cLTP induction and for unstimulated controls as described above. In the control, the proportion of the different shapes was mainly conserved over the time course (Figure 3B). Approximately 93% of the PSD95 assemblies were of macular shape before cLTP at −15 min, 90% at 60 min, and 91% at 120 min. Similarly, the proportions of perforated, segmented 2, and segmented ≥3 PSD95 assemblies was maintained in the control. However, in the case of potentiated spines, the proportion of macular PSD95 assemblies was 91% before cLTP induction, but decreased to 86% at 30 min, 78% at 60 min, and 73% at 120 min after cLTP (Figure 3B). This reduction of macular shapes was accompanied by an increase of segmented 2 and perforated PSD95 assemblies. For instance, the number of segmented 2 was 7% before cLTP and increased up to 12% at 30 min, 16% at 60 min and 17% at 120 min. Only 2% of the PSD95 assemblies were perforated before cLTP. This value increased to 4% after 60 min and 8% at 120 min after stimulation (Figure 3B). Altogether, this time-course shows that cLTP induction affects the PSD95 nano-pattern where PSD95 is reorganized into more complex, perforated, and segmented shapes after cLTP induction.

To quantitatively assess the reorganization of PSD95, we computed the coverage ratio of PSD95 in the synapse, which represents a measure for the degree of reorganization. To calculate the coverage ratio, we estimated the extent of the synapse by encircling all PSD95 of a synapse with an ellipse. Then we divided the area covered with PSD95 by the area of the ellipse (Figure 3C). Thus, the more black pixels there are between PSD95 segments or the larger a perforation is, the lower the coverage ratio. Analysing the coverage ratio for the whole time-course showed that it was essentially constant for all controls (Figures 3D and S5); its median value varied in ACSF treated controls between 0.78 (IQR 0.71–0.85) before cLTP at −15 min, 0.75 (IQR 0.70–0.81) at 60 min and 0.76 (IQR 0.68–0.85) at 120 min after cLTP (Figure 3D, left). The coverage ratio in potentiated spines exhibited a significant median decrease from 0.79 (IQR 0.73–0.88) before cLTP to 0.73 (IQR 0.65–0.82) at 60 min and to 0.73 (IQR 0.66–0.77) at 120 min after cLTP induction (Figure 3D, right). This supports the notion that the size expansion of PSD95 at 60 min after cLTP (Figure 2G) is accompanied by a remodeling of the PSD95 nano-organization.

### Synaptic GluA2 containing AMPA receptor area increases in size after chemically induced long-term potentiation

**Figure 4:**
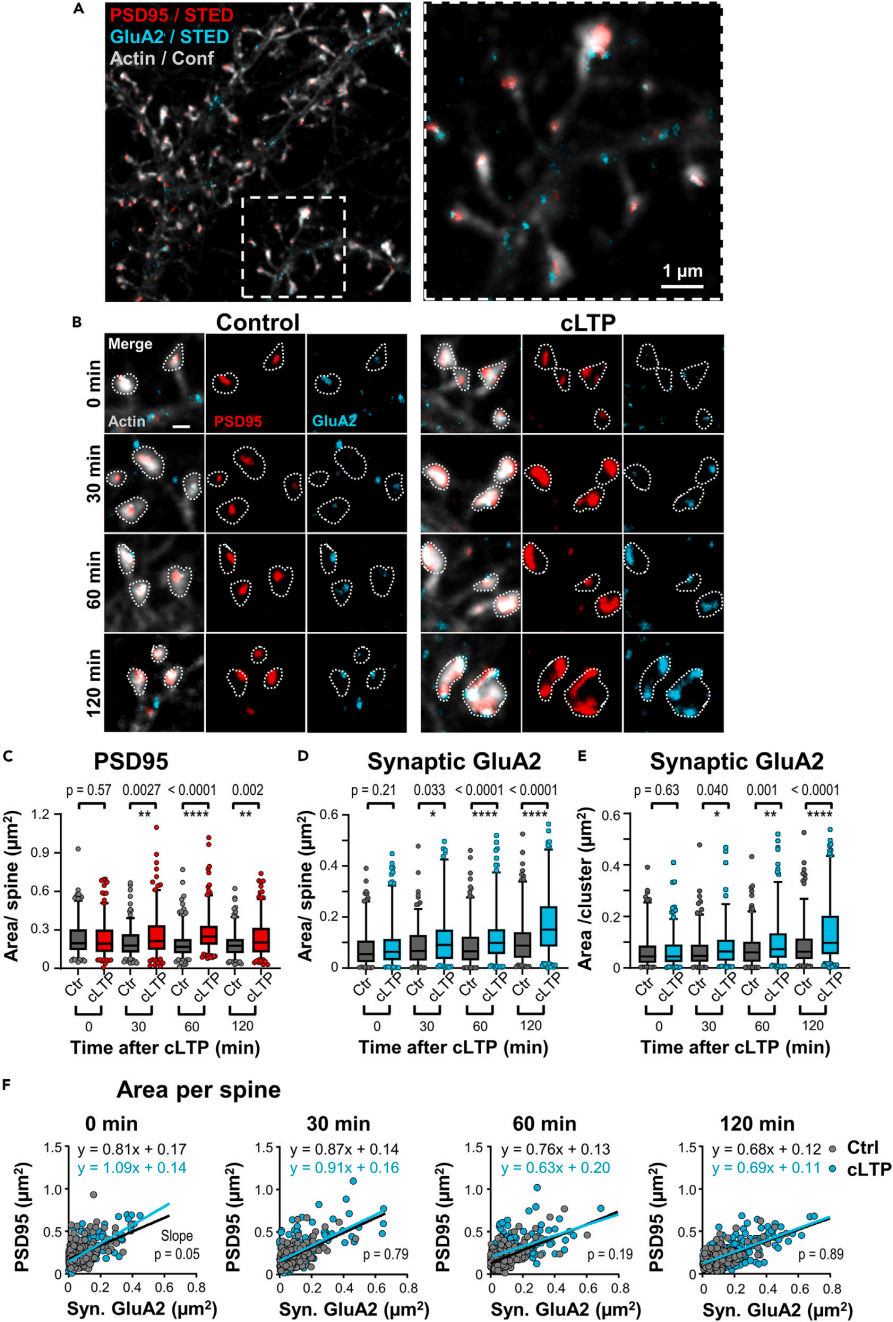
AMPAr nanocluster containing the GluA2 subunit and PSD95 nanostructures increase similarly in size after cLTP induction (A) Two-color STED image of PSD95 and GluA2 (immunolabelling), and confocal image of F-actin (labeled with phalloidin) in hippocampal neuronal cell culture at 17 DIV. (B) Time series of hippocampal neuronal cultures fixed at 0, 30 min, 60 min, and 120 min after cLTP induction (right) or without stimulation (control, left). Scale bar: 500 nm. (C) Median of PSD95 area per spine head; box and whisker plot with 25%–75% percentiles (box) and 5%–95% percentiles (whisker) (Mann-Whitney test). (D) Median and box and whisker plot of total GluA2 area per synapse following cLTP induction and of control (Mann-Whitney test). (E) Median and box and whisker plot of the area of single synaptic GluA2 nanocluster with and without cLTP induction (Mann-Whitney test). (F) Correlation between the size of the PSD95 and synaptic GluA2 area at different time points after cLTP induction or control samples fixed at the same time points; line shows linear regression. Pearson’s correlation coefficient r for control/cLTP: 0 min: 0.47/0.68; 30 min: 0.60/0.64; 60 min: 0.61/0.57; 120 min: 0.61/0.68. No significant difference in slope (p value displayed). (C–F) Data did not pass D'Agostino-Pearson normality test. Number of analyzed spines: Control: 0 min: 188, 30 min: 199, 60 min: 222, 120 min: 188; cLTP: 0 min: 207, 30 min: 181, 60 min: 220, 120 min: 213. Spines were analyzed per condition from at least 7 fields of view from 3 independent experiments. Source data: Table S4.

Next, we examined whether the activity-driven plasticity of PSD95 would also affect the nano-organization of AMPAr. An increase in the number of functional postsynaptic AMPAr is regarded as a fundamental mechanism of LTP.^21^ AMPAr consist of tetramers of four different subunits, GluA1–GluA4, which can be labeled specifically. In hippocampal neurons, most AMPAr contain the GluA2 subunit, which we therefore chose to label.^22^ Since there is no live-cell-compatible marker for GluA2 we employed immunohistochemistry to label GluA2 in fixed, cultured neurons at different time points after cLTP induction. At the same time, we immunolabeled PSD95 and tagged the F-actin cytoskeleton with fluorescence-labeled phalloidin (Figure 4A). cLTP was induced for 5 min and thereafter the cells were transferred to standard ACSF and fixed either immediately (0 min) or after 30, 60, or 120 min. Control cells were transferred at the same time points between ACSF solutions and fixed at the same time points. With a home-built microscope, the immunolabeled PSD95 and GluA2 were recorded in super-resolution STED and actin was detected in confocal mode (Figure 4B). The size of the GluA2 and PSD95 nanostructures was assessed by encircling the outer extent of the nano-pattern (Figure S6A). We only considered synaptic GluA2, i.e., we only included GluA2 in the spine head, which touches or overlaps with the PSD95 nano-pattern. The areas of separate cluster in a spine head were summed to obtain the PSD95 or synaptic GluA2 area per spine head. After cLTP induction the area of synaptic GluA2 per spine head increased significantly from 0.056 (median; 0.046/0.072 lower/upper 95% CI) μm^2^ at 0 min to 0.079 (0.064/0.098) μm^2^ after 30 min, to 0.098 (0.078/0.111) μm^2^ after 60 min and to 0.150 (0.129/0.179) μm^2^ after 120 min (Figures 4D and S6E). At the same time a significant increase in area was observed for PSD95 after 30 min, 60 min and 120 min (Figures 4C and S6D); for example, after 60 min the PSD95 assembly size increased from 0.169 (median; 0.154/0.181 lower/upper 95% CI) μm^2^ for control to 0.249 (0.228/0267) μm^2^ after cLTP induction. This increase shifts and broadens the frequency distribution of PSD95 assembly and synaptic GluA2 sizes (Figures S6D and S6E) similar to that of the mEPSC amplitudes (Figure 2C). The median size of GluA2 assemblies is on average 30% of the respective PSD95 assembly size in control samples and 50% of the PSD95 assembly size upon cLTP induction, i.e., they do not cover the entire PSD95 assembly. Plotting the sizes of PSD95 assemblies vs. synaptic GluA2 assemblies revealed a moderate to strong correlation with a Pearson’s correlation coefficient *r* of 0.6–0.7 for all time points after cLTP induction and in the control measurements (Figure 4F). Interestingly, the linear regression line of PSD95 area vs. synaptic GluA2 area is always relatively similar between cLTP and control (Figure 4F), indicating that PSD95 and synaptic GluA2 areas grow by a similar factor at each time point. Remarkably, the regression line has a relatively large positive y-intersect at all time points (Figure 4F). This suggests that tiny GluA2 nanoclusters are located on PSD95 assemblies that are ∼ two times larger in size than the GluA2 nanocluster itself, whereas large GluA2 nanoclusters, e.g., with a size of 0.5 μm^2^, appear on PSD95 assemblies of a similar size on average.

Next, we tested whether the increase of the synaptic GluA2 area was due to an enlargement of existing clusters or the appearance of additional GluA2 nanoclusters at the synapse. Synaptic GluA2 containing nanoclusters increased significantly in size after 30 min and continued to increase after 60 and 120 min after stimulation (Figure 4E). However, we also found a small increase in the number of GluA2 containing nanoclusters per synapse, which was significant at 0 and 120 min after cLTP induction (Figure S6C). Thus, we mainly observed an increase in the GluA2 nanocluster size, which might be accompanied by an increase in the number of clusters per synapse. Of note, not all spine heads did contain GluA2. Spines without AMPAr at the synapse cannot be activated and are therefore called silent synapses. Supporting previous findings,^11^^,^^16^ we observed that the number of putatively silent synapses dropped over our time course; 120 min after cLTP induction only 1.4% of the synapses were putatively silent compared to 6.9% without cLTP (Figure S6F). This corroborates the notion that LTP promotes the gradual insertion of new GluA2 at silent synapses.

### Collective pre- and postsynaptic enlargement

**Figure 5:**
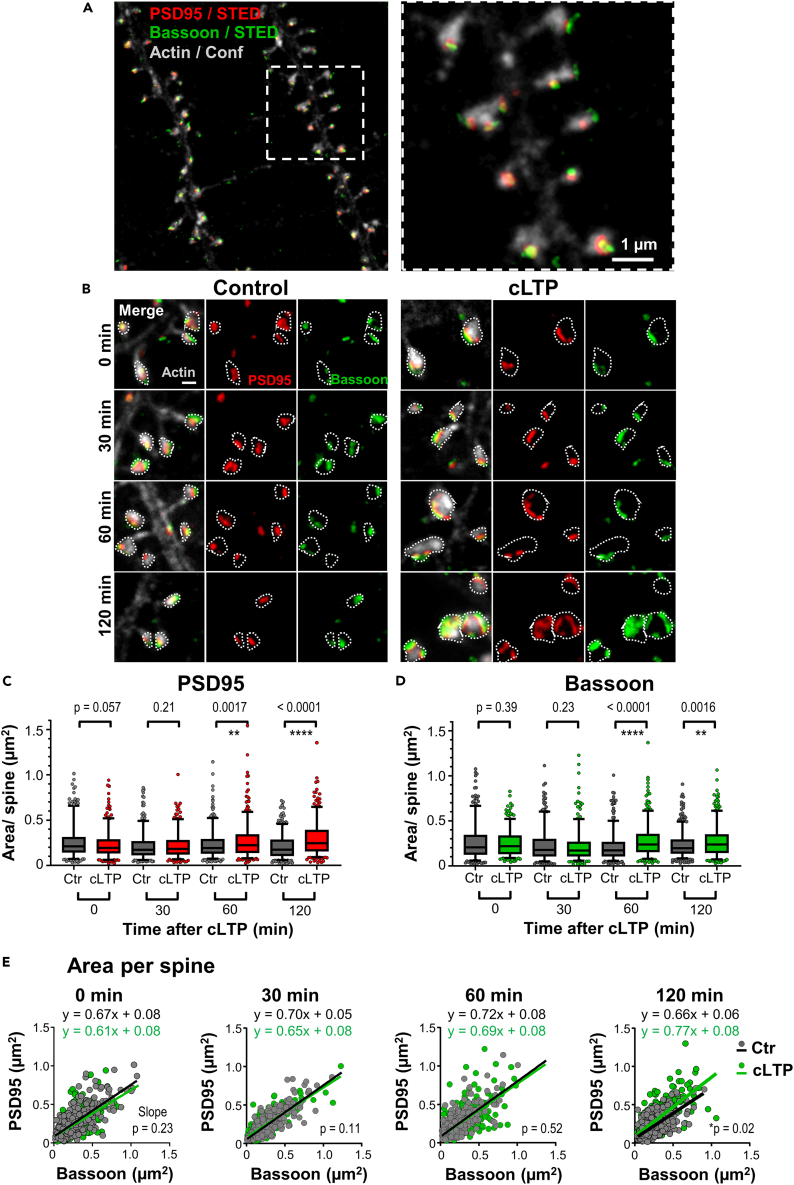
Coordinated increase of PSD95 and Bassoon assembly size after cLTP induction (A) Two-color STED microscopy of PSD95 and Bassoon (immunohistochemistry labeling), and confocal image of F-actin (phalloidin labeling) in a hippocampal neuronal culture at 17 DIV. (B) Time series of neurons fixed at 0, 30 min, 60 min, and 120 min after cLTP induction (right) or control samples fixed at the same time points without stimulation (left). Scale bar: 500 nm. (C) PSD95 assembly area per spine at 0, 30 min, 60 min, and 120 min after cLTP induction compared to control (median, box and whisker plot; Mann-Whitney test). (D) Same as (C), but for Bassoon area facing PSD95 (Mann-Whitney test). (E) Correlation between PSD95 and Bassoon assembly area per spine at 0, 30 min, 60 min, and 120 min following cLTP compared to control; line shows linear regression. Pearson’s correlation coefficient r for control/cLTP: 0 min: 0.75/0.58; 30 min: 0.83/0.79; 60 min: 0.74/0.67; 120 min: 0.68/0.71. (C–E) Data did not pass D'Agostino-Pearson normality test. Number of analyzed spines: Control: 0 min: 359, 30 min: 381, 60 min: 421, 120 min: 466; cLTP: 0 min: 340, 30 min: 310, 60 min: 424, 120 min: 370. Spines were analyzed per condition from at least 7 fields of view from 3 independent experiments. Source data: Table S5.

Recently, it has been shown that pre- and postsynaptic elements align to form so-called molecular nanocolumns.^9^^,^^10^ Therefore, the activity-induced enlargement and remodeling of PSD95 assemblies and AMPAr nanoclusters might be accompanied by corresponding presynaptic changes. Thus, we tested whether the size and nano-pattern of the presynaptic active zone protein Bassoon follows the same tendency as the PSD95 organization after cLTP induction. We fixed cultured hippocampal neurons at different time-points (0, 30, 60, and 120 min) after 5 min of cLTP induction (Figure 5B) as described above. We immunolabeled the neurons with antibodies against Bassoon and PSD95, and the F-actin cytoskeleton with phalloidin. Super-resolution imaging of Bassoon and PSD95 with STED microscopy resolved their synaptic nano-pattern and showed an increase in size following cLTP (Figures 5A and 5B). The area covered by Bassoon and PSD95 was analyzed by encircling the assemblies analogous to the analysis of GluA2 clusters. Only Bassoon assemblies that either overlapped with PSD95 or were directly opposite and thus most likely part of a synaptic contact were considered. After cLTP induction, Bassoon assemblies changed in size from 0.218 (median; 0.197/0.238; lower/upper 95% CI) μm^2^ directly after cLTP (0 min) to 0.168 (0.155/0.177) μm^2^ after 30 min, increased up to 0.238 (0.213/0.256) μm^2^ after 60 min, and to 0.236 (0.212/0.259) μm^2^ after 120 min. While the first two time points were not significantly different from the control group, the size of Bassoon assemblies increased significantly 60 min and 120 min after cLTP (Figure 5D). PSD95 assemblies also increased in size after 60 and 120 min (Figure 5C); for example, after 120 min they increased from 0.174 (0.161/0.190) μm^2^ in controls to 0.244 (0.225/0.270) μm^2^ after cLTP. Thus, the average size of Bassoon and PSD95 assemblies was very similar. The linear regression line of PSD95 area vs. Bassoon area was similar between cLTP and control groups at 0, 30, and 60 min after stimulation (Figure 5E); consequently, Bassoon and PSD95 assembly areas increase by the same factor on average after cLTP induction. However, the PSD95 assembly area increased slightly more than that of Bassoon after 120 min, which is indicated by a slightly larger slope of the regression line (Figure 5E). In summary, the changes in size of the pre- and postsynaptic scaffolding proteins Bassoon and PSD95 upon cLTP are largely correlated during the remodeling for up to 120 min after cLTP induction.

### Synaptic nano-organization of PSD95, GluA2, and Bassoon and its plasticity

**Figure 6:**
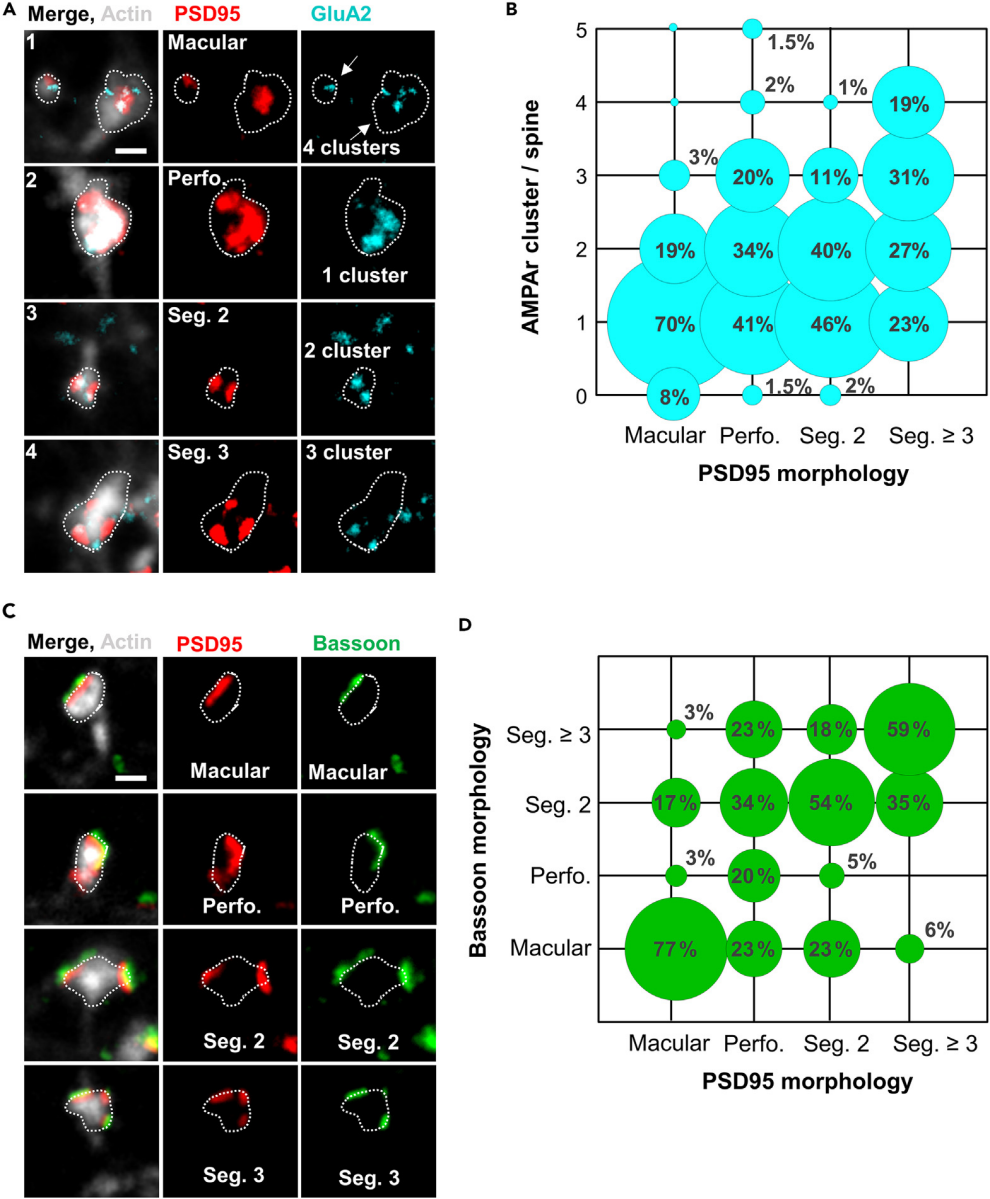
Similar nanoarchitecture across the synaptic scaffolds and AMPA receptors (A) Categorization of PSD95 nano-organization (red) and GluA2 containing AMPAr nanocluster (blue). PSD95 structures are categorized into macular, perforated, or assemblies which consist of 2 or 3 separated segments; GluA2 categorized in number of nanoclusters located on PSD95 per spine. For an overview image refer to Figure S7A. Scale bar: 500 nm. (B) Frequency of the number of AMPA receptor clusters as function of PSD95 morphologies; all time points and with and without cLTP induction pooled. Source data: Table S4. (C) Examples of the different PSD95 morphologies and presynaptic Bassoon nanostructures. Scale bar: 500 nm. (D) Frequency of Bassoon morphologies on different PSD95 nano-organizations. Source data: Table S5. Number of analyzed spines (B) are the same as in Figure 4 and for (D) the same as in Figure 5; cLTP, control and all time points were pooled.

Our time-lapse imaging of PSD95 in living, hippocampal slice cultures revealed a reorganization associated with an increase in the complexity of the PSD95 nano-pattern after cLTP induction (Figure 3B). Similarly, we also observed an increase in perforated and segmented PSD95 assemblies after cLTP induction in fixed, immunolabeled, cultured neurons (Figures 6, S7A, and S7B). As described for the time-lapse experiment, we categorized the PSD95 nano-pattern into macular, perforated, segmented 2, and segmented ≥3. At 60 and 120 min after cLTP induction, the distribution of the PSD95 nano-organization was significantly different from the control condition (Figure S7B). At 120 min after stimulation, the proportion of macular PSD95 decreased to 59% compared to 82% in the control, the proportion of perforated PSD95 increased to 22% compared with 3% in the control, and the proportion of segmented 2 also increased to 18% compared with 12% in the control. In summary, the structural change of PSD95 assemblies from macular to segmented and/or perforated ones occurs with a delay after cLTP induction and was found in both organotypic hippocampal slices and dissociated hippocampal neuronal cultures.

We next examined whether the postsynaptic nano-organization of PSD95 correlates with its corresponding presynaptic nanostructure and/or AMPAr clusters. Therefore, we analyzed the nanostructure of GluA2 and Bassoon assemblies accross all time points, with and without cLTP stimulation in relation to their associated PSD95 nanostructure (Figure 6). The GluA2 nano-organization, an important functional indicator of cLTP, was much less complex than that of PSD95; we did not observe perforations, and therefore only assigned cluster numbers for GluA2 nano-organizations (Figure 6A). Most of the synapses contained one GluA2 nanocluster, and about one-quarter of the synapses contained two clusters (Figure S7C). Figure 6B shows the frequency of the number of GluA2-containing nanoclusters as a function of the morphology of the associated PSD95 assemblies. This plot reveals, for example, that macular PSD95 mostly contain only a single GluA2 nanocluster. Perforated PSD95 nanostructures, on the other hand, contain one, two, or three GluA2 nanocluster with similar frequency. Nearly half of the segmented 2 PSD95 assemblies were occupied with only one GluA2 nanocluster, indicating that only one of the two PSD95 segments contained a GluA2 receptor patch. Similarly, ∼50% of the segmented 3 PSD95 contained only one or two GluA2 nanoclusters, indicating that not every segment contained GluA2. Interestingly, putatively silent synapses without GluA2 occurred almost exclusively on macular PSD95.

The nanostructure of Bassoon was similar to that of PSD95, so we also classified it as macular, perforated, segmented 2, and segmented ≥3 (Figure 6C). We pooled all data with and without cLTP stimulation and found a strong similarity between associated PSD95 and Bassoon nanostructures (Figure 6D). For example, macular Bassoon was associated with macular PSD95 assemblies in 77% of the cases, segmented 2 Bassoon with segmented 2 PSD95 and segmented 3 Bassoon with segmented 3 PSD95 in >50% of all cases. On perforated PSD95, however, Bassoon occurred in all different nanostructures with similar frequency. The similarity in the nanostructure of PSD95 and Bassoon assemblies is also reflected in the frequency of their appearance (Figure S7D). Overall, these data document a high correlation and similarity between pre- and postsynaptic scaffolding proteins regarding their structural organization at the nanoscale which is in line with proposed nanocolumn organization.

## Discussion

In this study we used super-resolution time-lapse STED microscopy to explore activity-dependent synaptic plasticity based on changes in the size and nanostructure of assemblies of three major building blocks of glutamatergic synapses; the pre- and postsynaptic scaffolding proteins, Bassoon and PSD95, and the ionotropic glutamate receptor, AMPAr. All three protein assemblies increased slowly in size and complexity over the entire imaging period of 2 h after cLTP induction; this enhancement was much slower than the rapid increase in synaptic strength as measured by mEPSCs events or the spine head growth. The strong correlation between the structural changes of the pre- and postsynaptic scaffold protein assemblies and their temporal progression triggered by synaptic activity implied a subtle tuning between these structural parameters.

In the past, many studies showed a tight correlation between the size of the spine volume and the PSD.^23^ Accordingly, the two parameters are often regarded as equivalent landmark indicators of changes in synaptic strength. In our measurements, however, the spine head increased already within 10 min of cLTP induction, while the increase in PSD95 assembly size was much slower, peaking at ∼60 min after cLTP induction. This is in line with electron microscopy^2^ and super-resolution microscopy^7^^,^^8^ studies reporting a temporal delay of the PSD95 assembly size increase after stimulation. This temporal decoupling may also explain why we found only a weak correlation between changes in PSD95 assembly and spine head size in mice after enhanced synaptic activity.^6^ However, a temporal decoupling of the correlation does not argue against a correlation at equilibrium or after adaptation. Interestingly, the relative increase in PSD95 assembly size was less than that of the spine head. Taken together, this indicates that PSD95 assembly and spine head size are each modulated by different mechanisms.

Our super-resolution imaging of endogenous PSD95 corroborates the complex shape of PSD95 assemblies that we often observed before, also *in vivo.*^5^^,^^6^ This shape is so diverse that a simple classification into clusters, as often done in the context of super-resolution microscopy,^3^^,^^11^ may underestimate their intricate function. We found that the complexity of PSD95 assemblies increased markedly after cLTP stimulation, peaking at the end of our measurement series of 2 h; for example, ∼18% of the macular PSD95 assemblies transformed into a more complex shape with a perforation or split into two or more segments 2 h after cLTP induction (Figure 3B). We detected a complex shape of PSD95 assemblies less frequently than in a previous study.^2^ This could be due to the fact that our super-resolution imaging in 2D cannot resolve the nanostructure along the z axis, which may underestimate the complexity of the structures. However, this limitation on super-resolution along the z axis does not affect the timing of the changes or the differences we describe here.

As previous studies indicated a strong structural and molecular coordination across the synapse in so-called *trans*-synaptic nanocolumns,^9^ we performed a size and shape analysis of presynaptic Bassoon after cLTP induction. Assemblies of presynaptic Bassoon and postsynaptic PSD95 were very similar in size and shape; the size increase of Bassoon assemblies peaked at ∼ 60 min after stimulation, i.e., at a similar timescale as PSD95 assemblies. As such, the structural correlation between PSD95 and Bassoon assemblies was even stronger than proposed previously.^9^ Thus, our data support the notion that synaptic activity can modulate a *trans*-synaptic structural correlation and alignment of molecular components. Intensive efforts are underway to determine how these structures align with each other across the synaptic cleft.^24^^,^^25^

A hallmark of LTP is the rapid potentiation of AMPAr-mediated postsynaptic currents and spine enlargement, which occur within a few minutes or less.^26^^,^^27^ Current hypotheses pose that AMPAr are highly dynamic within the plasma membrane around the synapse and are trapped at synapses.^21^ A remaining question is whether solely an increase in postsynaptic receptor number or also changes in the nanoscale organization of receptors contribute to changes in synaptic efficacy.^28^ In particular, the low affinity of AMPAr for glutamate requires a tight alignment between AMPA receptors and glutamate release sites,^3^^,^^25^ emphasizing a potential role of the synaptic nanoarchitecture. Previous studies indicate that AMPAr indeed align with presynaptic release sites^9^ and that AMPAr become more clustered at the periphery of the PSD, whereas NMDA receptors are more likely to cluster at its center.^29^ To investigate now activity-dependent changes of AMPAr, we super-resolved the nano-organization of GluA2 at different time points after cLTP induction. In line with the current literature, we found a clustered distribution of GluA2 within the synapse. Specifically, we did not observe complex structures such as perforations for GluA2, indicating a different nano-architecture for GluA2 assemblies as compared to for PSD95 or Bassoon assemblies. The median size of the GluA2 assemblies was about 1/3 of that of PSD95 assemblies, i.e., synaptic GluA2 was restricted to a sub-region of PSD95 assemblies. Thus, the trapping of receptors by PSD95 does not occur at stochastically random PSD95 molecules, but involves an additional, as yet unknown mechanism that causes clustering in subdomains within PSD95 assemblies. After cLTP induction, the synaptic GluA2 nanoclusters increased highly significantly in size and slightly in number, which deviates from a previously described purely modular architecture.^10^^,^^11^ The increase in area size of synaptic GluA2 continued for up to 2 h after cLTP induction and was therefore much slower than the reported rapid increase in AMPAr mediated currents after glutamate uncaging^26^^,^^27^ and also slower than the increase in the mEPSC quantal sizes that we observed after cLTP induction (Figure 2B). Therefore, our data do not indicate that the initial, rapid increase in mEPSC quantal sizes is due to an increase in synaptic GluA2 levels. However, this initial increase may be mediated by a reorganization of synaptic AMPAr and a targeting with presynaptic release sites without a change in number of AMPAr.^30^ In essence, the essential characteristics of LTP formation cannot be explained only by changes AMPA receptor number.

The scaled increase in the mEPSC amplitude (Figures 2C and S2) and the increase and widening of the size distribution of GluA2 and PSD95 assemblies (Figures S6D and S6E) indicate that the synaptic strengthening involves most synapses and not just a few specific synapses. The strong, positive y-intersect of the linear regression line in Figure 4F, fitted to the plot of PSD95 assembly sizes versus synaptic GluA2, indicates that the size ratio between PSD95 and GluA2 assemblies is different between small and large PSD95 assemblies. Thus, larger synapses with larger PSD95 assemblies harbor proportionally more GluA2. This is consistent with the hypothesis that larger synapses may have experienced synaptic strengthening and therefore integrated more AMPAr.^21^^,^^31^

Currently, the most convincing model for the formation of synaptic nanostructures is that of liquid-liquid phase separation.^32^ Indeed, reconstituted PSDs were shown to self-organize into a web-like structure similar to native perforated PSD.^32^^,^^33^ Moreover, CaMKII is able to drive the segregation of AMPA and NMDA receptors into separate nanodomains within the PSD.^34^ Although these models so far include only a small selection of PSD proteins, they may become adequate to describe the formation of synaptic nanostructures in the future.

### Limitations of the study

It is fundamental for this study to use neuronal and organotypic cultures which are healthy and of good quality. It is important to choose the correct labeling schema to avoid labeling artifacts. We chose PSD95.FingR for the live-cell experiment to label endogenous PSD95 and tested different fixation methods and concentrations according to^35^ to optimize the specificity of the immunocytochemistry.

## STAR★Methods

### Key resources table

**Table:** 

REAGENT or RESOURCE	SOURCE	IDENTIFIER
**Antibodies**		

rabbit anti-bassoon	Synaptic Systems	Cat# 141013; RRID: AB_2744651
mouse anti-PSD95	Neuromab	Cat# 75-028; RRID: AB_2877189
Monoclonal mouse anti-GluA2	Millipore	Cat# MAB397; RRID: AB_2113875
rabbit anti-PSD95	Cell signaling	Cat# 3450; RRID: AB_2292883

**Chemicals, peptides, and recombinant proteins**		

Ara-C	Sigma-Aldrich	Cat# C6645, CAS# 69-74-9
Uridine	Sigma-Aldrich	Cat# U3750, CAS# 58-96-8
5-Fluoro-2′-desoxyuridine	Sigma-Aldrich	Cat# F0503, CAS#50-91-9
glycine	Sigma-Aldrich	Cat# G8790
bicuculline	Hello Bio	Cat# HB0893, CAS# 40709-69-1
D-2-amino-5-phosphonovaleric acid (APV)	Hello Bio	Cat# HB0225
glyoxal	Sigma-Aldrich	Cat# 128465
Goat serum	Sigma-Aldrich	Cat# G9023
Triton X-100	Sigma-Aldrich	Cat# T8787
Alexa Fluor 488 phalloidin	Invitrogen	Cat# A12379
tetrodotoxin (TTX)	Tocris	Cat# 1078

**Experimental models: Organisms/strains**		

Mouse: C57BL/6J	JAX	RRID:IMSR_JAX:000664

**Recombinant DNA**		

rAAV-hSyn-DIO-myr-rsEGFP2-LDLR(ct)-WPRE	Willig et al., *Cell Rep* 35, 109192 (2021)^13^	N/A
rAAV-ZFN-hSyn-DIO-PSD95.FingR-Citrine-reg.-WPRE	Willig et al., *Cell Rep* 35, 109192 (2021)^13^	N/A
rAAV-hSyn-CRE-WPRE	Wegner et al., *Sci Rep* 7, 11781 (2017)^19^	N/A

**Software and algorithms**		

Fiji/ImageJ	Schindelin et al., 2012	RRID:SCR_002285
GraphPad Prism	GraphPad	RRID:SCR_002798
Imspector Software	Abberior Instruments	RRID:SCR_015249

**Other**		

Polytetrafluoroethylene membrane, pore size 0.45 μm	Millipore	Cat# FHLC01300
Cell culture insert of 0.4 μm pore size	Millipore	Cat# PICM03050

### Resource availability

#### Lead contact

Further information and requests for resources and reagents should be directed to and will be fulfilled by the Lead Contact Katrin I. Willig (e-mail: kwillig@mpinat.mpg.de).

#### Materials availability

Requests for plasmids should be directed to and will be fulfilled by the lead contact.

#### Data and code availability

• Source data files are attached as supplementary tables. Image datasets reported in this paper will be shared by the lead contact upon request.
• No code was generated.
• Any additional information required to reanalyze the data reported in this paper is available from the lead contact upon request.

### Experimental model and subject details

#### Animals

Mice and rats for breading were kept at the animal facility of the Max Planck Institute for Multidisciplinary Sciences, City Campus, in Göttingen and housed with a 12 h light/dark cycle, with food and water available *ad libitum*. Experiments were performed according to the guidelines of the national law regarding animal protection procedures and institutional permission was granted.

#### Neuronal hippocampal cultures

Primary neuronal hippocampal cell cultures were prepared according to^36^ from the hippocampi of Wistar rats of both sex at postnatal day 1 (P1). Cells were plated at density of 180,000 cells/ml on 18 mm round coverslips of thickness #1.5 (Marienfeld, # 0117580) in 12-well plates and maintained at 37°C with 5% CO_2_.

#### Organotypic hippocampal slices

Organotypic hippocampal slice cultures were prepared from P5 C57BL/6 wild-type mice of both sex according to.^37^ In brief, mice were decapitated and the hippocampus was extracted and sliced into coronal sections of 300 μm thickness. The slices were placed on small pieces of Polytetrafluoroethylene (PTFE) membrane, pore size 0.45 μm (Millipore, # FHLC01300) and cultured on cell culture insert of 0.4 μm pore size (Millipore, # PICM03050) placed in a 6-well plate and then incubated at 37°C with 5% CO_2_ for 18 to 23 days *in vitro* (DIV). The inhibitor mix containing Ara-C (Sigma-Aldrich, #C6645), Uridine (Sigma-Aldrich, #U3750) and 5-Fluoro-2′-desoxyuridine (Sigma-Aldrich #F0503), was added to a final concentration of 3 μM on the third day and the medium was exchanged three times per week.

### Method details

#### Live-cell labeling with rAAV

The membrane of dendrites and spine are labeled by transduction with rAAV-hSyn-DIO-myr-rsEGFP2-LDLR(ct)-WPRE^13^ which encodes for the reversible photoswitchable (rs) green FP rsEGFP2.^15^ The rsEGFP2 tag is attach to an N-terminal myristoylation motif (myr) that promoted membrane labeling, and the C-terminal (Ct) cytoplasmic domains of low-density lipoprotein receptor (LDLR) to target the protein to the dendrite.^38^ The expression is cre-dependent by insertion of a double-floxed inverted open reading frame (DIO) under the control of the neuron-specific human synapsin-1 promoter (hSyn).

Endogenous PSD95 is visualized by transducing rAAV-ZFN-hSyn-DIO-PSD95.FingR-Citrine-reg.-WPRE^6^^,^^13^ encoding for a transcriptionally regulated recombinant antibody-like-protein called FingR (Fibronectin intrabodies generated with mRNA display) fused to the yellow FP Citrine.^14^

Both rAAV were transduced together with a low concentration of the cre-recombinase encoding virus rAAV-hSyn-CRE-WPRE^19^ in the CA1 region of an organotypic hippocampal slices 2 days after preparation. The virus was injected via a pulled needle of a borosilicate glass capillary (ID: 0.68mm, OD: 1.2mm; Kwik-fill, World Precision Instruments Inc., # 1B150F-4) that was angled at 50° to the slice using a stereotaxic micromanipulator (SM-11, (Narishige Scientific Instrument Lab.). ∼50 nL of the virus mixture was pressure injected with ∼10 pulses at 15 psi via an Intracellular Microinjection Dispense System (PICOSPRITZER III, Parker Instrumentation).

#### Chemical LTP

For immunostaining experiments, hippocampal cultured neurons were used between 16 and 21 DIV. To induce cLTP, we treated the neurons for 5 min at 37°C with modified artificial cerebrospinal fluid (ACSF) in which magnesium was removed, and 200 μM glycine (Sigma-Aldrich, #G8790) and 20 μM bicuculline (Hello Bio, # HB0893) were added instead.^39^ The composition of ACSF for the hippocampal cultured neurons was as follows (mM); 2 MgCl_2_, 105 NaCl, 2,4 KCl, 10 HEPES, 10 D-glucose, 2 CaCl_2_ at pH 7.4, and ∼240 mOsm. The treated neurons were immediately fixed (time point 0 min) or kept in ACSF and fixed after 30, 60, or 120 min post cLTP induction. As control, the hippocampal cultured neurons were incubated in ACSF and fixed at the same time points.

To record the cLTP with live-cell STED imaging of hippocampal organotypic slices, we also used the modified ACSF for 10 min. However, the composition of the ACSF used here is as follows (mM); 2 MgCl_2_, 128 NaCl, 2 KCl, 10 KH_2_PO_4_, 26 NaHCO_3_, 10 glucose, 2 CaCl_2_, pH 7.4 and ∼320 mOsm. Before induction of cLTP, the slices were maintained in ACSF solution supplemented with 50 μM D*-*2-amino-5-phosphonovaleric acid (APV, Hello Bio, # HB0225) for 20 min to block the activity mediated by NMDA receptors. After cLTP induction, the slices were perfused with ACSF for 2 h. For control, the solutions were changed at the same time points but all solutions contained ACSF supplemented with 50 μM APV for blocking (marked as APV throughout the manuscript). To investigate the basal activity, the hippocampal organotypic slices were maintained in the ACSF during the whole experiment (marked as CONTROL). During the live-STED imaging sessions, the hippocampal slices were preserved at 30°C via a heated platform (QE-2, Warner Instruments, LLC) and solution heater (SF-28, Warner Instruments, LLC); both are controlled by a dual temperature controller (TC-344C, Warner Instruments, LLC). The ACSF solution was continuously infused with 5% CO_2_ and delivered with a flow of ∼1 mL/min (MINIPULS 3 Peristaltic Pump, Gilson).

#### Immunocytochemistry

The cultured hippocampal neurons were fixed in a 3% v/v glyoxal solution (Sigma-Aldrich, # 128465) containing 0.75% acetic acid (Carl Roth, # 3738) for 1 h according to.^35^ The fixative was adjusted to pH 4 for staining Bassoon/PSD95 and to pH 5 for staining GluA2/PSD95. After fixation, cells were quenched in 0.1 M glycine in PBS and then permeabilized for 30 min in a blocking solution of 2% normal goat serum (Sigma-Aldrich, #G9023) and 0.1% Triton X-100 (Sigma-Aldrich, #T8787) in PBS. The primary antibodies rabbit anti-bassoon (Synaptic Systems, # 141013, dilution 1:500), mouse anti-PSD95 (Neuromab, # 75-028, dilution 1:300), and Alexa Fluor 488 phalloidin (Invitrogen, # A12379, 1:600) were diluted in blocking solution and the neurons were incubated for 2 h at room temperature.

To label AMPA receptors in the plasma membrane the N-terminal extracellular domain of GluA2 was stained with monoclonal mouse anti-GluA2 (Millipore, # MAB397, 1:500) for 2 h without prior permeabilization. Thereafter, cells were permeabilized in blocking solution supplemented with 0.1% Triton X-100 for further intracellular labeling of PSD95 and actin. The samples were incubated with rabbit anti-PSD95 (Cell signaling, # 3450, 1:300) and Alexa Fluor 488 phalloidin (1:600) diluted in blocking solution (with Triton X-100) for 2 h at room temperature.

After washing, the secondary antibodies anti-rabbit STAR RED (Abberior, # STRED-1002, dilution 1:50) and anti-mouse Alexa Fluor 594 (Thermo Fisher Scientific, # A-11005, dilution 1:100) were incubated at 4°C overnight in blocking solution. Finally, the coverslips were mounted with Mowiol (Carl Roth, # 0713).

#### Live-cell STED imaging of brain slices

For live-cell imaging of the organotypic hippocampal brain slices the 2D STED microscope was configured as describe in^13^ with the following minor adaptions. The images were collected with a water dipping objective of 1.2 numerical aperture equipped with a correction collar (HC PL APO 63x/1.20 W CORR CS2, Leica Germany, # 506356). Fluorescence of both, rsEGFP2 and Citrine, was detected between 510 and 560 nm by a bandpass filter (AHF Analysentechnik). Images of PSD95 (Citrine) and the membrane label (rsEGFP2) were recorded sequentially. Firstly, PSD95 was superresolved using the blue light excitation together with the STED beam at 595 nm; immediately afterward, a confocal image of the myristoylation tag was recorded at the same dwell-time and excitation power, without the STED beam, but with additional UV light to switch the rsEGFP2 to the on state.^13^ The switching was very fast and therefore no additional switching step was required; the rsEGFP2 was switched on during the readout of the fluorescence. See ref.^13^ for details.

The organotypic hippocampal slices were imaged at 18 to 23 DIV. Therefore, a slice was placed in a 35 mm Petri dish and attached to the bottom of the dish with silicon glue (twinsil, picodent, # 1300 1000) to prevent the slice from floating away or moving during time-lapse imaging. The slice was continuously perfused with ACSF and heated as described above. Confocal and 2D STED x,y-images were recorded of apical dendrites in the CA1 region in stacks over 2.5 μm at a distance Δz of 500 nm and with a dwell-time of 4 μs with the software Imspector (Abberior Instruments). The dimension of the x, y-image was 30 × 30 μm with a pixel size of 30 nm square. 2D STED super-resolution was achieved in the x, y plane only. Fast coarse confocal overview images were recorded before each STED image stack to realign the field of view with the earlier recorded image area. For time-lapse imaging STED and confocal stacks were acquired 4 times in two different settings: before cLTP, during cLTP, 30 and 60 min after cLTP, or before cLTP, 30, 60, and 120 min after cLTP. The power of the blue excitation beam was 5.5 μW, that of the UV light for switching 2 mW and STED was performed with ∼15 mW measured at the entrance pupil of the objective, respectively.

#### Dual-color 2D STED and confocal imaging of fixed cells

A home-built inverted dual-color 2D STED microscope for red emitting fluorescent dyes described in^19^ was slightly modified to image the triple color immunostaining of Bassoon, PSD95 and actin or respectively GluA2. A blue laser line was added to excite Alexa Fluor 488 at 488 nm with 8 μW (Cobolt 06-MLD, HÜBNER Photonics); its fluorescence was detected after a confocal pinhole in the range 510–560 nm. Super-resolution STED was performed on STAR RED, excited with 18 μW red light of 630 nm central wavelength and detected at 692/40 nm, and Alexa Fluor 594, excited with 15 μW in the orange at 586 nm and detected at 620/14 nm. Both, STAR RED and Alexa Fluor 594 were depleted at 775 nm with 230 mW power measured at the entrance pupil of the objective. All images were acquired quasi-simultaneously by repeating each line three times with alternated excitation wavelengths and detection channels. Images were collected in stacks of 5 pictures of 30 × 30 μm in x, y over 2 μm at distances Δz of 400 nm; the x/y pixel size was 20 nm squared and pixel dwell-time 5 μs.

#### Electrophysiological recording

Miniature excitatory postsynaptic current (mEPSC) was recorded in CA1 pyramidal neurons of organotyptic hippocampal slice culture at DIV 16–21 using the whole cell patch recording mode under voltage-clamp conditions at −70mV, using a double patch-clamp amplifier (EPC-10, HEKA) with Patchmaster software; the recording electrode (2.5–3.5 MΩ) contained internal solution (mM) with 138 K-gluconate, 16.8 HEPES, 10 NaCl, 1 MgCl_2_, 4 ATP-Mg, 0.3 GTP-Na and 0.25 K-EGTA at pH 7.38 and 310 mOsm. The external solution was carbogen-saturated ACSF containing (mM) 120 NaCl, 20 KCl, 10 KH_2_PO_4_, 26 NaHCO_3_, 10 glucose, 2 CaCl_2_, with a pH of 7.4 and 302 mOsm. The AAV-PSD95-FingR-Citrine and AAV-myr-rsEGFP2 were overexpressed in the neurons by the viral system at 2 DIV. The organotypic hippocampal slices were constantly supplied with the ACSF before and during the recording. For cLTP, we record the mEPSC before and after treatment with modified ACSF (Mg^2+^ free, 200 μM glycine and 20 μM bicuculline) which was applied for 10 min. After treatment, mEPSCs were recorded for another hour in the standard ACSF. All extracellular solutions for mEPSC were continuously infused with 1 μM tetrodotoxin (TTX) (Tocris, # 1078) and 20 μM bicuculline.

### Quantification and statistical analysis

#### Image processing

For the live-cell imaging experiments, the size of the spine head and corresponding PSD95 area were analyzed with Fiji/ImageJ.^40^ To ensure a standardized procedure, all images were acquired with the same laser power and all images were processed in the same way. The images were first smoothed by replacing each pixel with the average of its 3 x 3-pixel neighborhood, the standard routine in ImageJ, and the background was subtracted. To measure the spine head and PSD95 area each was encircled with the freehand selection tool. The changes in both PSD95 area (Δ PSD95 area) and spine head area (Δ spine head area) at each time point was calculated as ΔA/A_0_. The value A_0_ corresponds to the initial time point (before cLTP); ΔA refers to the difference between the areas at time point t (0, 30, 60 or 120 min after cLTP) with the initial time point A_0_. To normalize to control, the normalized control values were subtracted at each time point.

PSD95 nano-organizations were categorized according to their shape by a visual inspection. Spine heads containing only one PSD95 assembly without any perforation or nanostructure was assigned a macular shape. PSD95 with a U-shape, ring-like or more complex shape, which was continuously connected was assigned a perforated morphology; holes in the center or discontinuities were only considered if they were at least 3 x 3 pixels (90 nm × 90 nm) in size. When PSD95 occurred in more than one spot per spine head and these spots were separated by at least 3 pixels, the number of the segments was counted.

The spreading of PSD95 in a synapse was estimated by computing the coverage ratio. By that means, we estimated the extent of the synapse with an ellipse encircling the complex PSD95 assemblies. The coverage was computed by dividing the area covered with PSD95 by the area of the encircled ellipse, the outer expansion; thus $overage=\frac{PSD95\;area}{outer\;expansion}$. In this way the coverage drops the larger a hole in a perforation gets or the more two clusters are separated. The PSD95 area was obtained by encircling PSD95 with the freehand selection tool in FIJI; the area of the ellipse encircling the PSD95 assembly was obtained by fitting the length and the width of PSD95 assembly with an ellipse in Fiji.

The two-color STED images of immunolabeled PSD95 and GluA2 or Bassoon, respectively, were analyzed in Fiji as follows. The images were smoothed and the background was subtracted. The area was obtained by encircling each protein assembly manually. The size of segmented PSD95 in a spine head was summed. Bassoon was only included when it was contacting PSD95. The shape of the PSD95 and Bassoon nano-pattern was analyzed as described above and assigned a macular, perforated or segmented shape. GluA2 did not show perforations and thus only the number of clusters was counted; included were only clusters located on PSD95 nano-organizations, and clusters were required to be separated at least by 3 pixels (60 nm).

#### Statistical analysis

Repeated measures with randomly missing values were analyzed with mixed-effects analysis with Dunett’s multiple comparisons test. A Kruskal-Wallis with Dunn’s multiple comparisons test was performed for non-normally distributed data and more than two conditions. The data was tested for normality by the D'Agostino-Pearson test. The comparison between two conditions was performed with the Mann-Whitney test for non-normally distributed data and unpaired t-test for normally distributed data. Cumulative distributions were tested with the Kolmogorov-Smirnov test. A chi-square test was applied for categorical variables. The respective test and number of experiments and analyzed structures are listed in the figure legend. All statistical analyses were generated via GraphPad Prism.

## Supplemental information

Document S1. Figures S1‒S7

Table S1. Spine head and PSD95 assembly size before, during and after cLTP induction, with and without stimulation; source data, related to Figures 2F and 2G

Table S2. PSD95 assembly shape before, during and after cLTP induction, with and without stimulation; source data, related to Figure 3B

Table S3. PSD95 coverage ratio before, during and after cLTP induction, with and without stimulation; source data, related to Figure 3D

Table S4. PSD95 assembly area and shape, synaptic GluA2 area and number of clusters after cLTP induction, with and without stimulation; source data, related to Figures 4 and 6B

Table S5. PSD95 and Bassoon assembly area and shape after cLTP induction, with and without stimulation; source data, related to Figures 5 and 6D
