# Supplementary material for: Pre- and postsynaptic nanostructures increase in size and complexity after induction of long-term potentiation
Source: iScience. 2023 Dec 7;27(1):108679. doi: 10.1016/j.isci.2023.108679 (PMC10783556; doi:10.1016/j.isci.2023.108679)
Supplement: Document S1. Figures S1‒S7 [file mmc1.pdf]

iScience, Volume *27*

Supplemental information

Pre- and postsynaptic nanostructures increase in size and complexity after induction of long-term potentiation

Valérie Clavet-Fournier, ChungKu Lee, Waja Wegner, Nils Brose, JeongSeop Rhee, and Katrin I. Willig

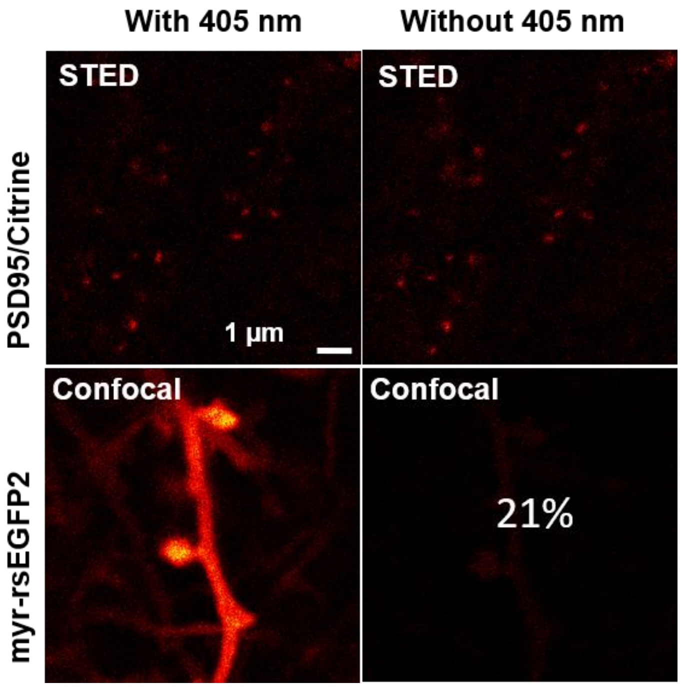

Figure S1: Imaging scheme, related to Figure 1. Neurons expressing either PSD95.FingR-Citrine (above) or myr-rsEGFP2 (below) in hippocampal organotypic slices. While both labels, Citrine and rsEGFP2, are excited at 480 nm, the latter can only be read out when it is switched to the on state by UV light (raw data); switching contrast between on and off state at the applied laser powers is 21 %.

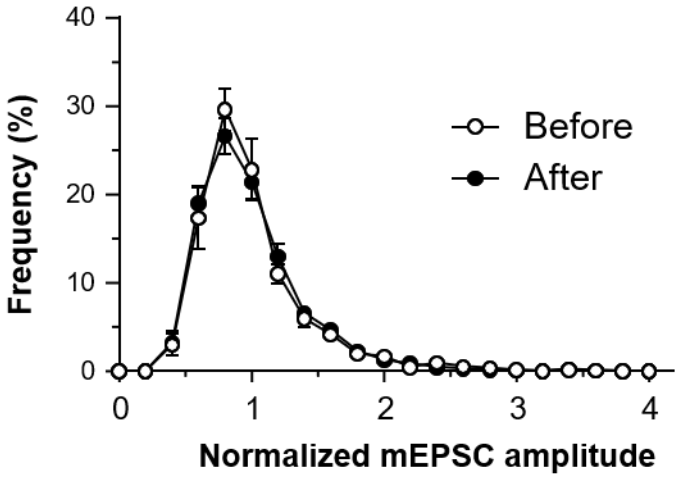

Figure S2: Frequency distributions of normalized mEPSC amplitudes, related to Figure 2. The distributions before (open circle, -15 min) and after cLTP induction (closed circle, 65 min) are very similar in shape. Same dataset as Figure 2C.

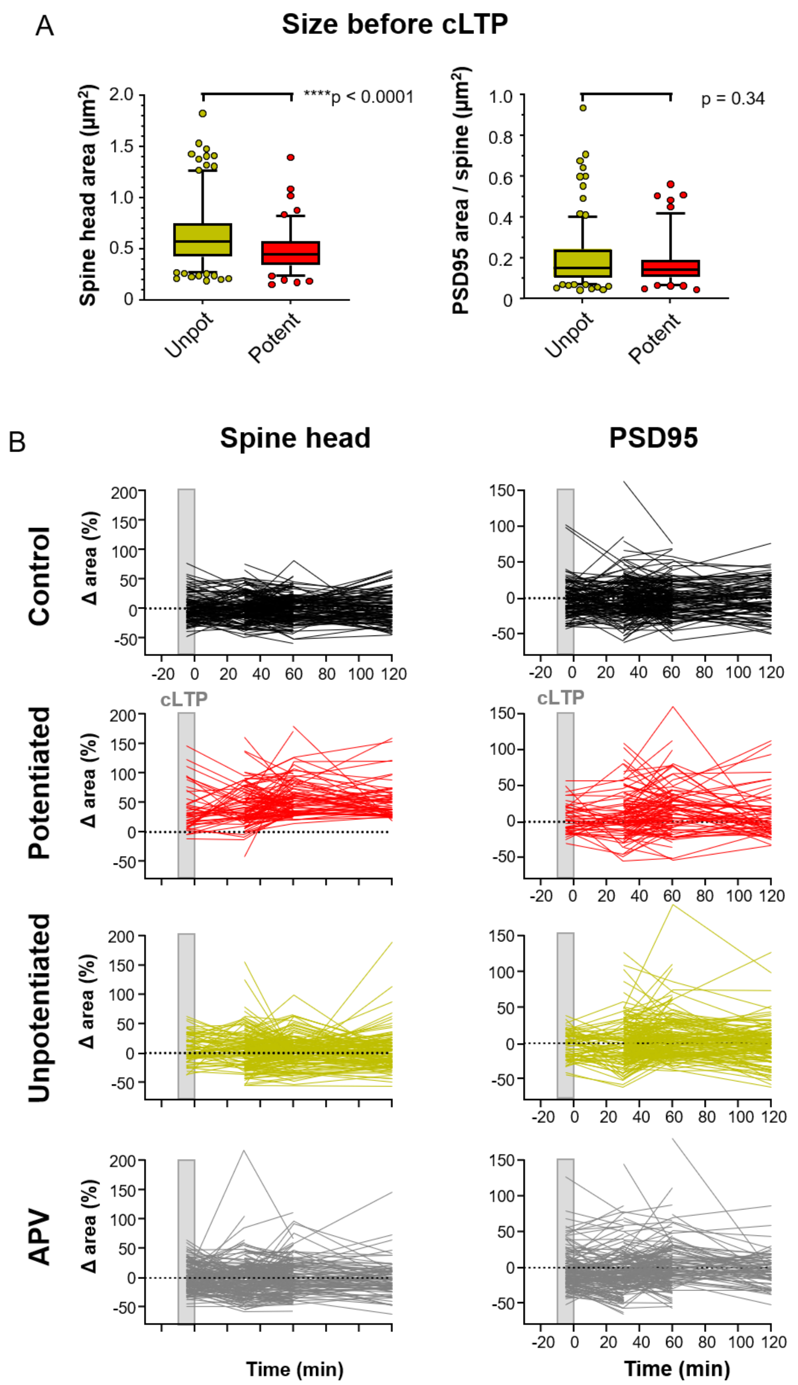

Figure S3: Size distribution before cLTP induction and size changes over time after stimulation, related to Figure 2. (A) Size distribution of spine heads (left) and PSD95 area per spine head (right) before treatment; median with box (25 % – 75 %, interquartile range, IQR) and whisker plot (5 % – 95 %). Median size of spine heads (IQR): Unpotentiated: 0.57 (0.43 – 0.75) µm^2; potentiated: 0.45 (0.34 – 0.58) µm^2. Median size of PSD95 assemblies: PSD95 on unpotentiated spines: 0.15 (0.10 – 0.24) µm^2; PSD95 on potentiated spines: 0.14 (0.11 – 0.19) µm^2 (Mann-Whitney test). **(B)** Changes in spine head area to before cLTP of potentiated and unpotentiated spines after cLTP relative to control; lines represent time-laps measurements of single spines. Control conditions were continuously kept in ACSF (control) or spines at blocked activity measured in ACSF containing APV (APV). Same data as in Figure 2F, G.

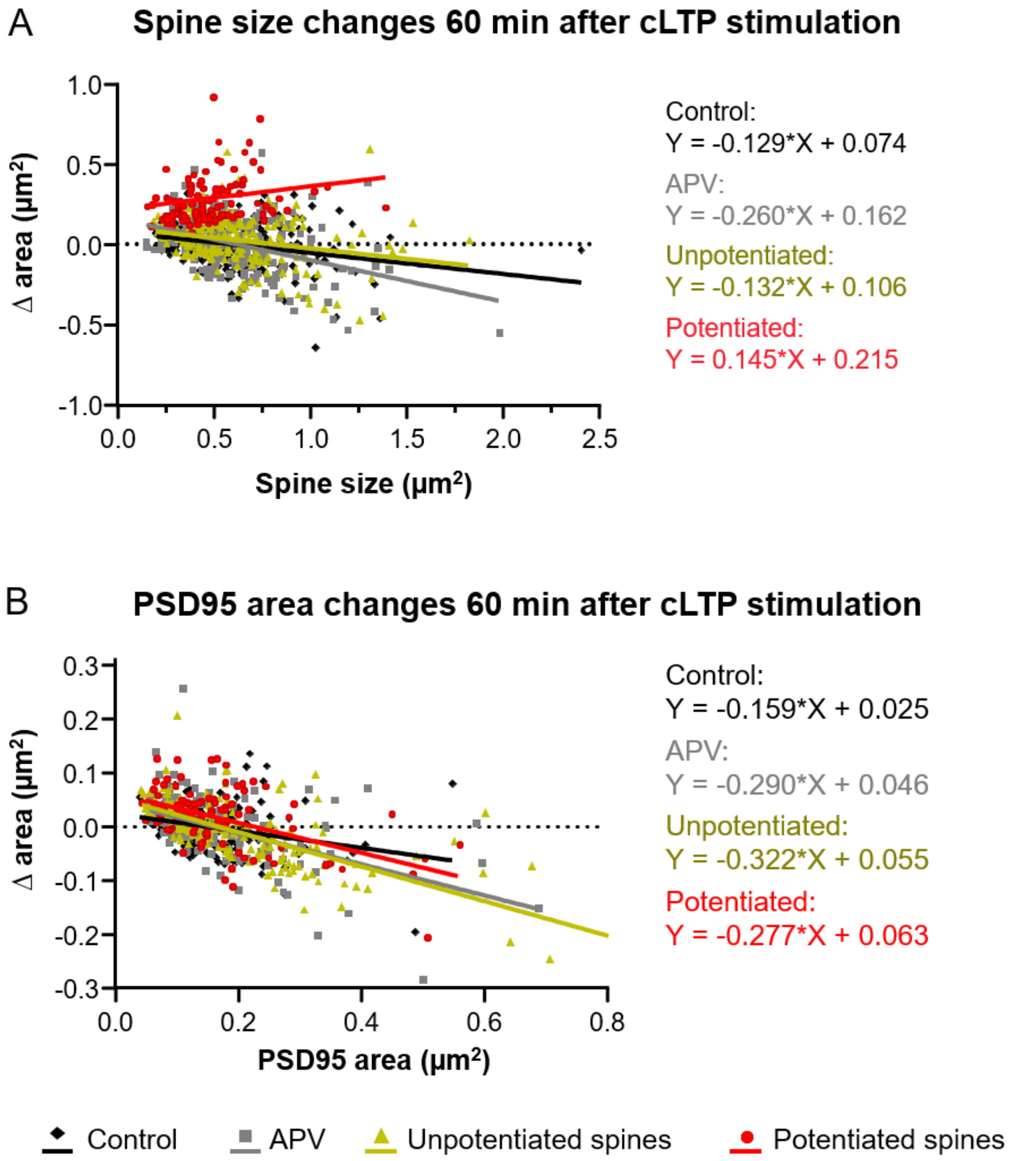

Figure S4: Changes of spine head and PSD95 assembly size at 60 min after cLTP induction depend on their initial size, related to Figure 2. (A) Changes of spine head size and **(B)** PSD95 area are plotted against their initial size before cLTP; straight lines are a linear regression of the corresponding data. Control, APV-treated and -unpotentiated spines and their PSD95 assemblies mainly show a regression to the mean effect, i.e. as such small spines or PSD95 assemblies, respectively, tend to grow while large ones tend to shrink. The regression line for potentiated spines (A, red) is higher, reflecting the increase in average spine size after cLTP induction; the change in slope from -0.129 for control to +0.145 indicates a multiplicative increase, i.e. the changes depend on the initial spine size. The regression line for the PSD95 area (B, red) is also slightly higher but overall similar to the control conditions.

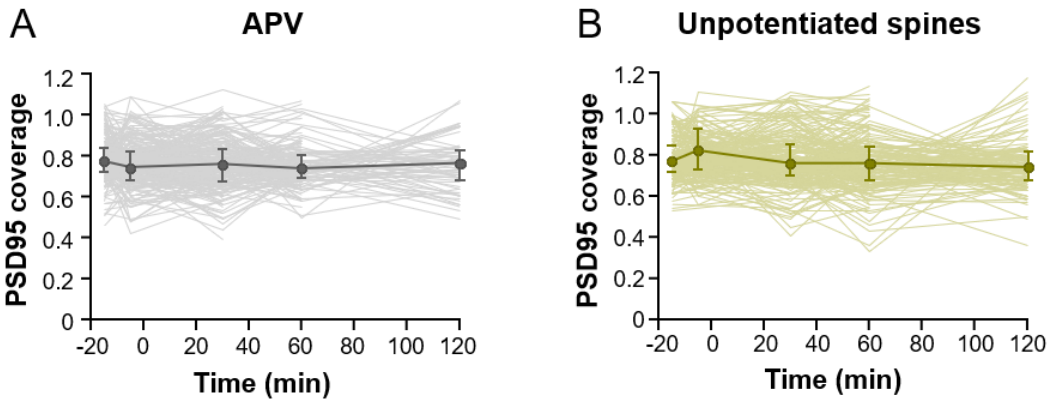

Figure S5: Time course of PSD95 coverage ratio, related to Figure 3. (A, B) PSD95 coverage ratio for APV treated (A) and unpotentiated spines (B) over a 120 min time course; single spine traces in light colours overlaid with median and IQR (Mixed-effects analysis with Dunett's multiple comparisons test; no significant changes over time).

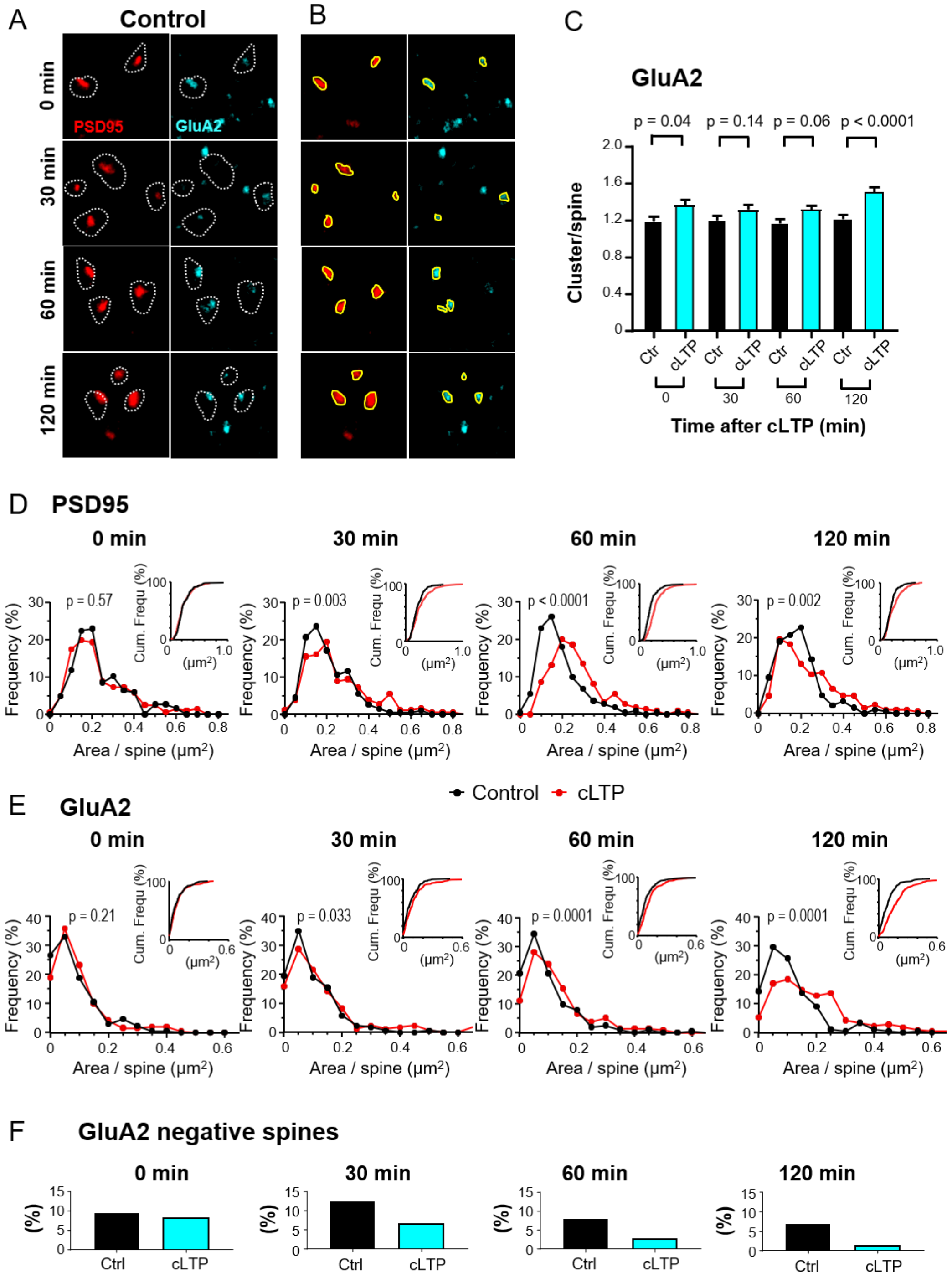

Figure S6: Size analysis of PSD95 assemblies and synaptic GluA2 after cLTP induction, related to Figure 4. (A, B) Size analysis of PSD95 assemblies and GluA2 containing AMPAr cluster exemplified on the control group of Figure 4; all assemblies per spine were outlined (B) to calculate the area covered. **(C)** The number of AMPA receptor (AMPAr) clusters per PSD95 nano-organization after cLTP induction compared to control; data shown as mean ± SEM (Mann-Whitney test). **(D, E)** Frequency distribution and cumulative frequency (inset) of PSD95 area (D) and GluA2 area (E) per spine; data, test and p value same as in Figure 4C, D. **(F)** Percentage of spines without AMPA receptor clusters based on PSD95 organizations following cLTP compared to control; 0 min, cLTP: 8.2 % vs Ctrl: 9.6 %; 30 min, cLTP: 6.6 % vs Ctrl: 12.6 %; 60 min, cLTP: 2.7 % vs Ctrl: 8.1 %; 120 min, cLTP: 1.4 % vs Ctrl: 6.9 %.

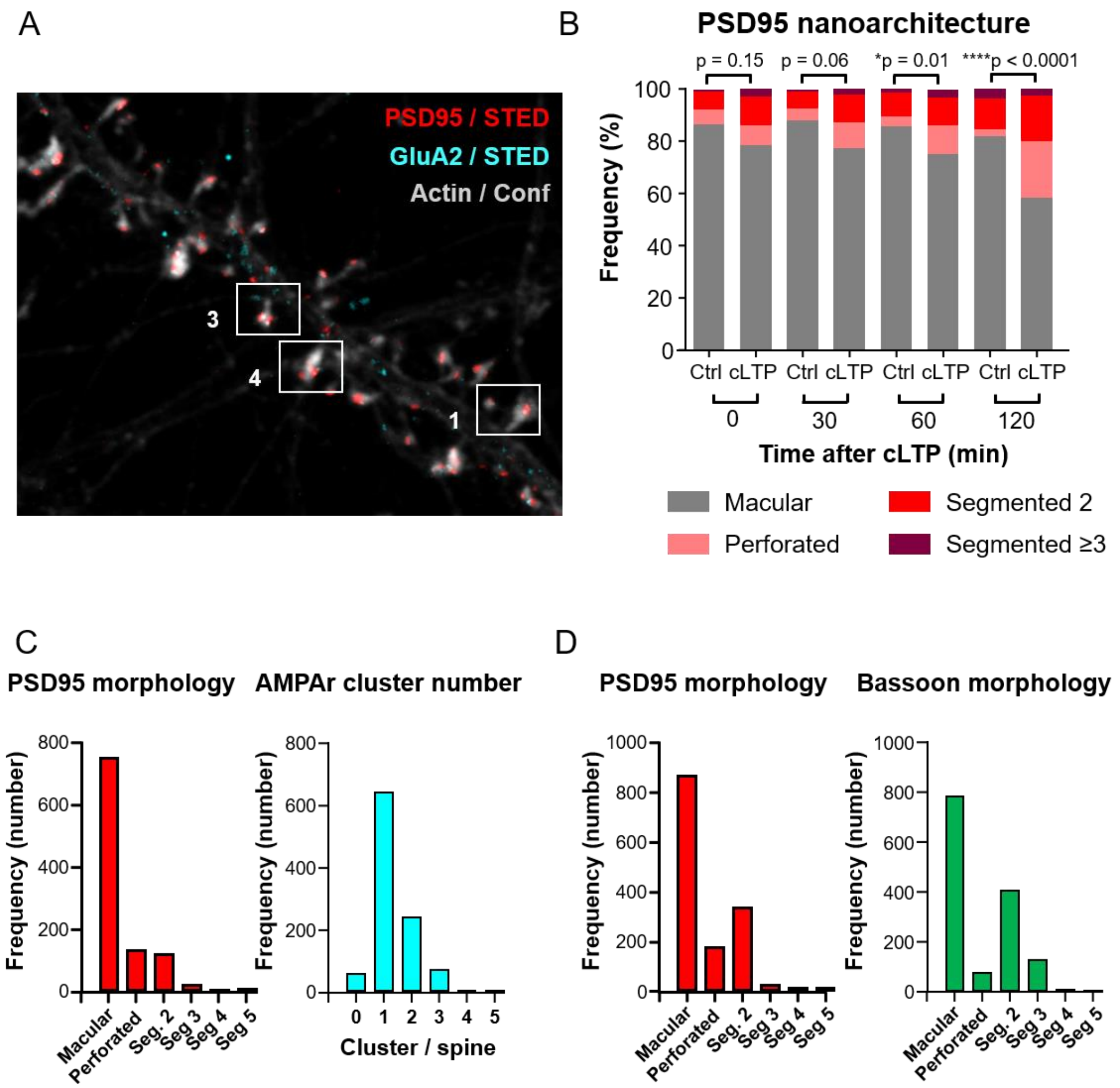

Figure S7: Synaptic nano-organization, related to Figure 6. (A) Overview image of PSD95, GluA2 and f-actin labeled neuronal culture; boxed and numbered are spines shown in Figure 6. **(B)** Frequency of PSD95 morphologies at 0, 30 min, 60 min, and 120 min following cLTP compared to control (Ctrl); the distribution of morphologies is significantly different between cLTP and control after 60 and 120 min (Chi-square test). (C) Frequency histogram of PSD95 nanostructure and number of synaptic GluA2 containing AMPAr nanocluster; all time points and conditions with and without cLTP induction were pooled; same data as Figure 6B. (D) Frequency histogram of PSD95 and Bassoon nanostructure; all time points and conditions pooled; same data as Figure 6D.
